# Supplementary material for: Molecular basis of accessible plasma membrane cholesterol recognition by the GRAM domain of GRAMD1b
Source: EMBO J. 2021 Feb 19;40(6):e106524. doi: 10.15252/embj.2020106524 (PMC7957428; doi:10.15252/embj.2020106524)
Supplement: Supplementary file 1 — Appendix [file EMBJ-40-e106524-s003.pdf]

Table of contents

Appendix Figure S1. Cholesterol- and anionic lipid-sensing mutations impair the PM recruitment of EGFP-$GRAM_{1b}$ **2**

Appendix Figure S2. GRAMD1b carrying an intellectual disability mutation (R189W) is defective in membrane tethering and cholesterol transport *in vitro* **3**

Appendix Figure S3. GRAMD1b carrying R191A and K161A/R191A mutations tether membranes and transport cholesterol efficiently *in vitro* **4**

Appendix Figure S4. The G187L mutation rescues the impaired PM recruitment of EGFP-$GRAM_{1b}$–R191A **5**

Appendix Figure S5. GRAMD1b carrying the G187L mutation extracts accessible PM cholesterol more efficiently than wild-type GRAMD1b **6**

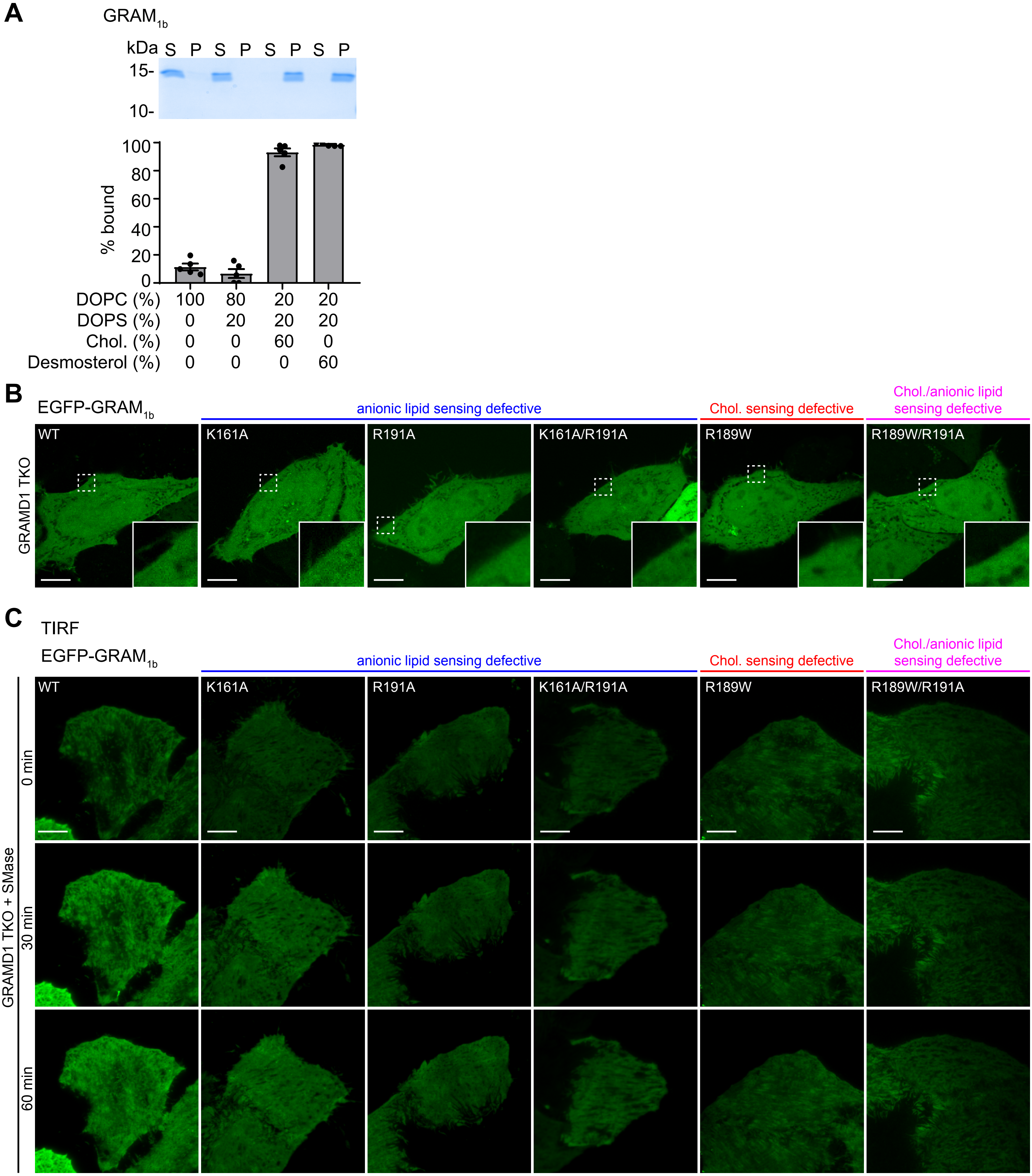

Appendix Figure S1. Cholesterol- and anionic lipid- sensing mutations impair the PM recruitment of EGFP-GRAM$_{1b}$

(A) Similar binding of GRAM$_{1b}$ to liposomes containing either cholesterol or desmosterol. Liposomes containing the indicated mole% lipids were incubated with purified GRAM$_{1b}$ proteins as shown. Bound proteins [pellet, (P)] were separated from the unbound proteins [supernatant, (S)], run on SDS-PAGE and visualized by colloidal blue staining (mean ± SEM, n = 5 independent experiments for all conditions).
(B) Confocal images of live GRAMD1 TKO HeLa cells expressing EGFP-GRAM$_{1b}$ constructs as indicated. Insets show at higher magnification the regions indicated by white dashed boxes. Scale bars, 10 μm.
(C) Snapshots of the cortical regions of live GRAMD1 TKO HeLa cells expressing indicated EGFP-GRAM$_{1b}$ constructs, imaged under TIRF microscopy, at different time points following SMase treatment (100mU/ml) as indicated are shown. Scale bars, 10 μm. See also **Movie EV1**.

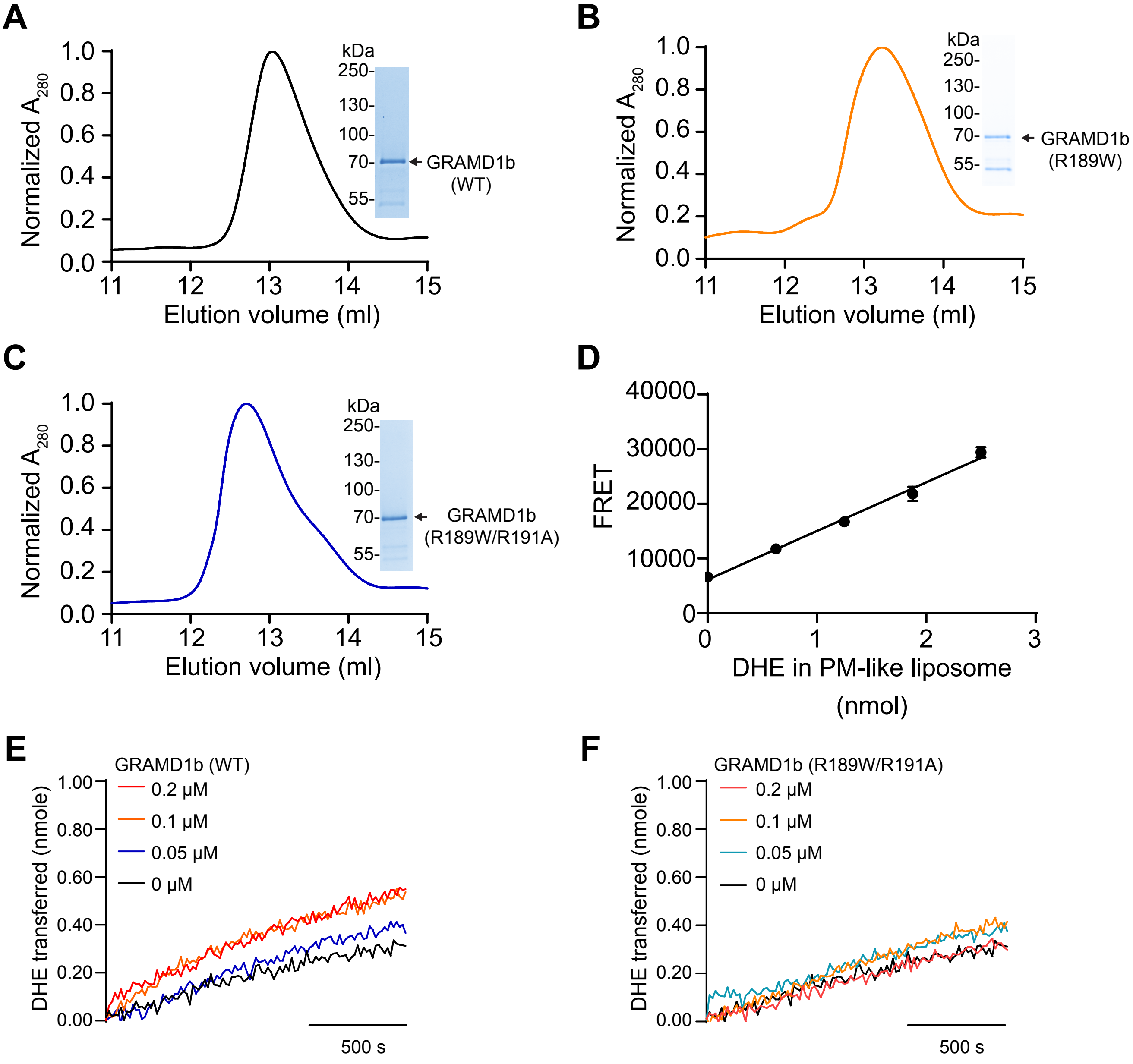

Appendix Figure S2. GRAMD1b carrying an intellectual disability mutation (R189W) is defective in membrane tethering and cholesterol transport *in vitro*

(A-C) Representative size exclusion chromatography (SEC) profiles of purified wild-type GRAMD1b proteins (WT) **(A)** and mutant GRAMD1b proteins, carrying either R189W mutation **(B)** or R189W/R191A double mutation **(C)**. The peak fraction was subjected to SDS-PAGE followed by colloidal blue staining.
(D) Calibration curve showing the linear relationship between FRET signals and the amount of DHE present in liposomes that contain 2.5 mole% of DNS-PE. This calibration curve was used to measure quantitatively the amount of DHE transferred from donor to acceptor liposomes over time (see Materials and Methods) (mean ± SEM, n = 3 for all different DHE concentrations).
(E-F) Representative time course of DHE transfer from L_{PM} to L_{ER} mediated by either wild-type GRAMD1b proteins (WT) **(E)** or mutant GRAMD1b R189W/R191A proteins (R189W/R191A) **(F)** as indicated. Purified GRAMD1 proteins (0.05 μM, 0.1 μM or 0.2 μM) were added at time 0. Addition of buffer alone (0 μM) was used as a control.

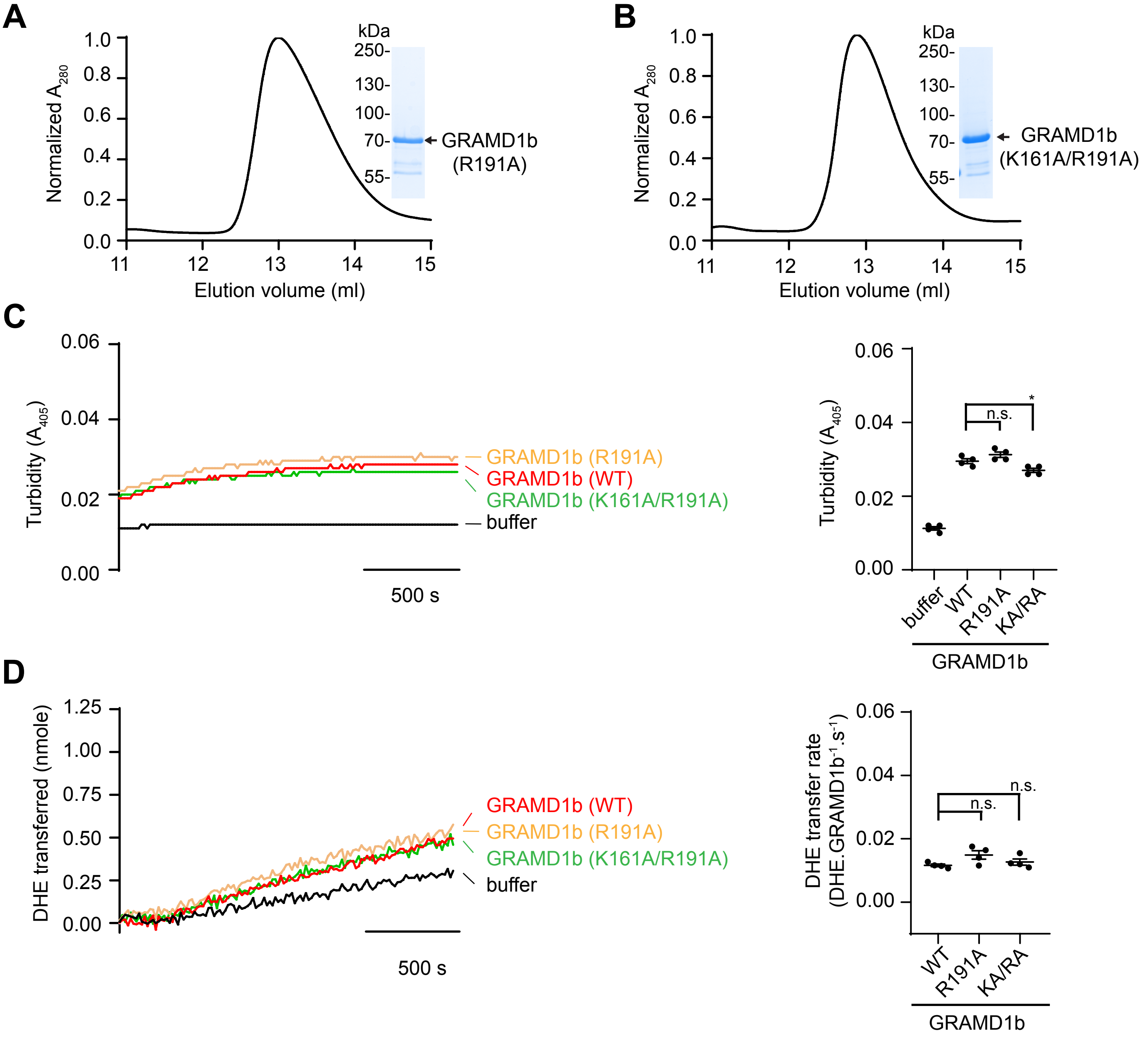

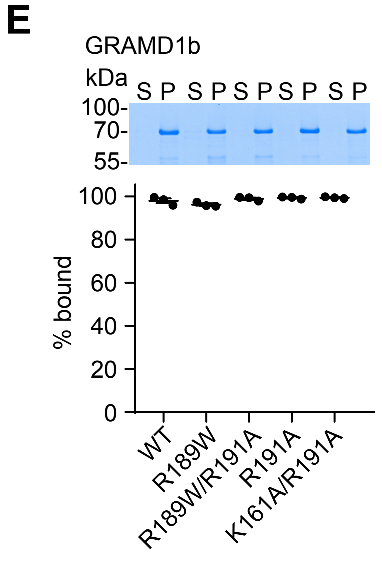

Appendix Figure S3. GRAMD1b carrying R191A and K161A/R191A mutations tether membranes and transport cholesterol efficiently *in vitro*

(A-B) Representative size exclusion chromatography (SEC) profiles of mutant GRAMD1b proteins, carrying either R191A mutation (A) or K161A/R191A double mutation (B). The peak fraction was subjected to SDS-PAGE followed by colloidal blue staining.
(C) Liposome tethering by purified GRAMD1b proteins. Left: Representative time course of liposome tethering, as assessed by turbidity of mixtures containing both L_{PM} and L_{ER} together with either wild-type GRAMD1b proteins (WT), mutant GRAMD1b R191A proteins (R191A), mutant GRAMD1b K161A/R191A proteins (KA/RA) or buffer alone as indicated. Experiments and data analysis were performed as in **Fig 4**. Right: Values of turbidity corresponding to the end of the experiment as indicated by the arrow (mean ± SEM, n = 4 independent experiments; Dunnett's multiple comparisons test, *P = 0.0373; n.s. denotes not significant).
(D) DHE transfer between liposomes by purified GRAMD1b proteins. Left: Representative time course of DHE transfer from L_{PM} to L_{ER} mediated by either wild-type GRAMD1b proteins (WT), mutant GRAMD1b R191A proteins (R191A), mutant GRAMD1b K161A/R191A proteins (KA/RA) or buffer alone as indicated. Experiments and data analysis were performed as in **Fig 4**. Right: Values of DHE transfer rate of GRAMD1b (WT), GRAMD1b (R191A), and GRAMD1b (KA/RA), as estimated by the FRET-based lipid transfer assay (mean ± SEM, n = 4 independent experiments; Dunnett's multiple comparisons test; n.s. denotes not significant).
(E) Liposome sedimentation assays of purified wild-type GRAMD1b proteins (WT) and mutant versions of GRAMD1b proteins carrying indicated GRAM domain mutations. Proteins were incubated with endoplasmic reticulum-like liposomes (L_{ER}) [15% DGS-NTA (Ni), 20% phosphatidylethanolamine (1-palmitoyl-2-oleoyl-sn-glycero-3-phosphoethanolamine/POPE), 65% DOPC]. Bound proteins [pellet, (P)] were separated from the unbound proteins [supernatant, (S)], run on SDS-PAGE and visualized by colloidal blue staining (mean ± SEM, n = 3 independent experiments for all conditions).

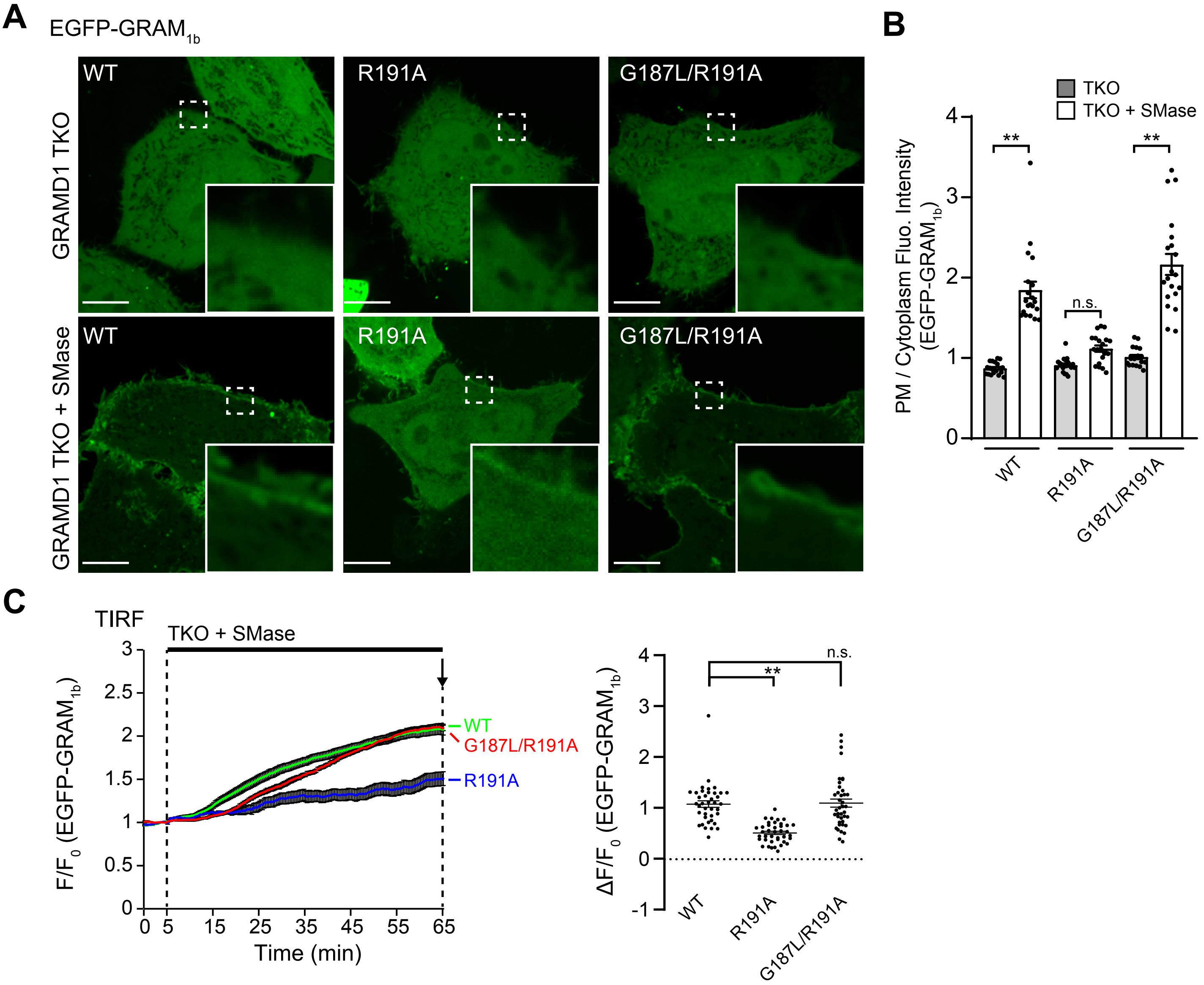

Appendix Figure S4. The G187L mutation rescues the impaired PM recruitment of EGFP-GRAM$_{1b}$–R191A

(A) Confocal images of live GRAMD1 TKO HeLa cells expressing either wild-type (WT) or mutant versions of EGFP-tagged GRAM domain of GRAMD1b (EGFP-GRAM$_{1b}$) constructs as indicated. Cells were treated with SMase (100 mU/ml for 1 hour at 37°C) before imaging. Insets show at higher magnification the regions indicated by white dashed boxes. Phosphatidylserine (PS) sensing defective mutant (R191A), and cholesterol hypersensitive and PS sensing defective double mutant (G187L/R191A) are shown. Note the significantly increased PM recruitment of EGFP-GRAM$_{1b}$ (G187L/R191A) compared to the reduced PM recruitment of EGFP-GRAM$_{1b}$ (R191A). Scale bars, 10 µm.

(B) Quantification of the ratio of PM EGFP-GRAM$_{1b}$ signals to the cytosolic EGFP-GRAM$_{1b}$ signals, as assessed by confocal microscopy and line scan analysis from GRAMD1 TKO HeLa cells expressing either wild-type (WT) or mutant versions of EGFP-GRAM$_{1b}$ with or without SMase treatment (100 mU/ml for 1 hour at 37°C) as shown in A [mean ± SEM, n = 20 cells for each condition; data are pooled from two independent experiments for each condition; two-tailed unpaired Student's t-test, **P < 0.0001. n.s. denotes not significant].

(C) Left: Time course of normalized EGFP signal, as assessed by TIRF microscopy, from GRAMD1 TKO HeLa cells expressing either wild-type (WT) or mutant versions of EGFP-GRAM$_{1b}$ constructs as indicated. SMase treatment (100 mU/ml) is indicated. Right: Values of ΔF/F0 corresponding to the end of the experiment as indicated by the arrow [mean ± SEM, n = 39 cells (WT), n = 39 cells (R191A), n = 40 cells (G187L/R191A); data are pooled from two independent experiments for each condition; Dunnett's multiple comparisons test, **P < 0.0001. n.s. denotes not significant].

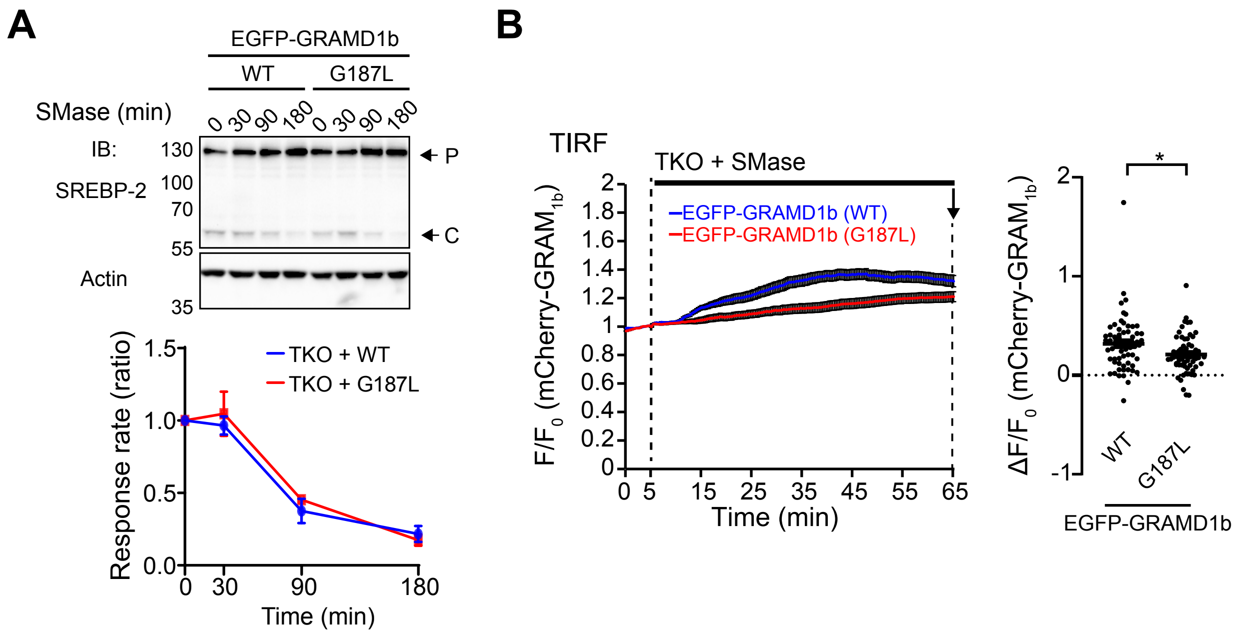

Appendix Figure S5. GRAMD1b carrying the G187L mutation extracts accessible PM cholesterol more efficiently than wild-type GRAMD1b

(A) GRAMD1 TKO (TKO) HeLa cells that stably expressed EGFP-tagged GRAMD1b constructs as indicated [wild-type (WT), G187L mutant (G187L)], were cultured in the medium supplemented with 10% lipoprotein-deficient serum (LPDS) and mevastatin (50 μM) for 16 hours and then treated with SMase (100 mU/ml) for indicated time at 37°C. Top: Lysates of the cells were processed for SDS-PAGE and immunoblotting (IB) with anti-SREBP-2 and anti-actin antibodies. Arrows indicate precursor (P) and cleaved (C) forms of SREBP-2. Bottom: The response rate was obtained by normalizing the ratio of the band intensity of the cleaved SREBP-2 over the total band intensity of cleaved and precursor forms of SREBP-2 from the cells with SMase treatment by the one from the cells without SMase treatment for each condition [mean ± SEM, n = 3 lysates (independent experiments) for each condition].
(B) Left: Time course of normalized mCherry signal, as assessed by TIRF microscopy, from GRAMD1 TKO HeLa cells stably expressing EGFP-GRAMD1b constructs as indicated that were additionally transfected with an accessible PM cholesterol biosensor mCherry-$GRAM_{1b}$. SMase treatment (100 mU/ml) is indicated. Right: Values of ΔF/F0 corresponding to the end of the experiment as indicated by the arrow [mean ± SEM, n = 63 cells (EGFP-GRAMD1b WT), n = 61 cells (EGFP-GRAMD1b G187L)]; data are pooled from four independent experiments for each condition two-tailed unpaired Student's t-test, *P = 0.0134)].
