# Supplementary material for: Molecular basis of accessible plasma membrane cholesterol recognition by the GRAM domain of GRAMD1b
Source: EMBO J. 2021 Feb 19;40(6):e106524. doi: 10.15252/embj.2020106524 (PMC7957428; doi:10.15252/embj.2020106524)
Supplement: Supplementary file 2 — Expanded View Figures PDF [file EMBJ-40-e106524-s007.pdf]

Expanded View Figures

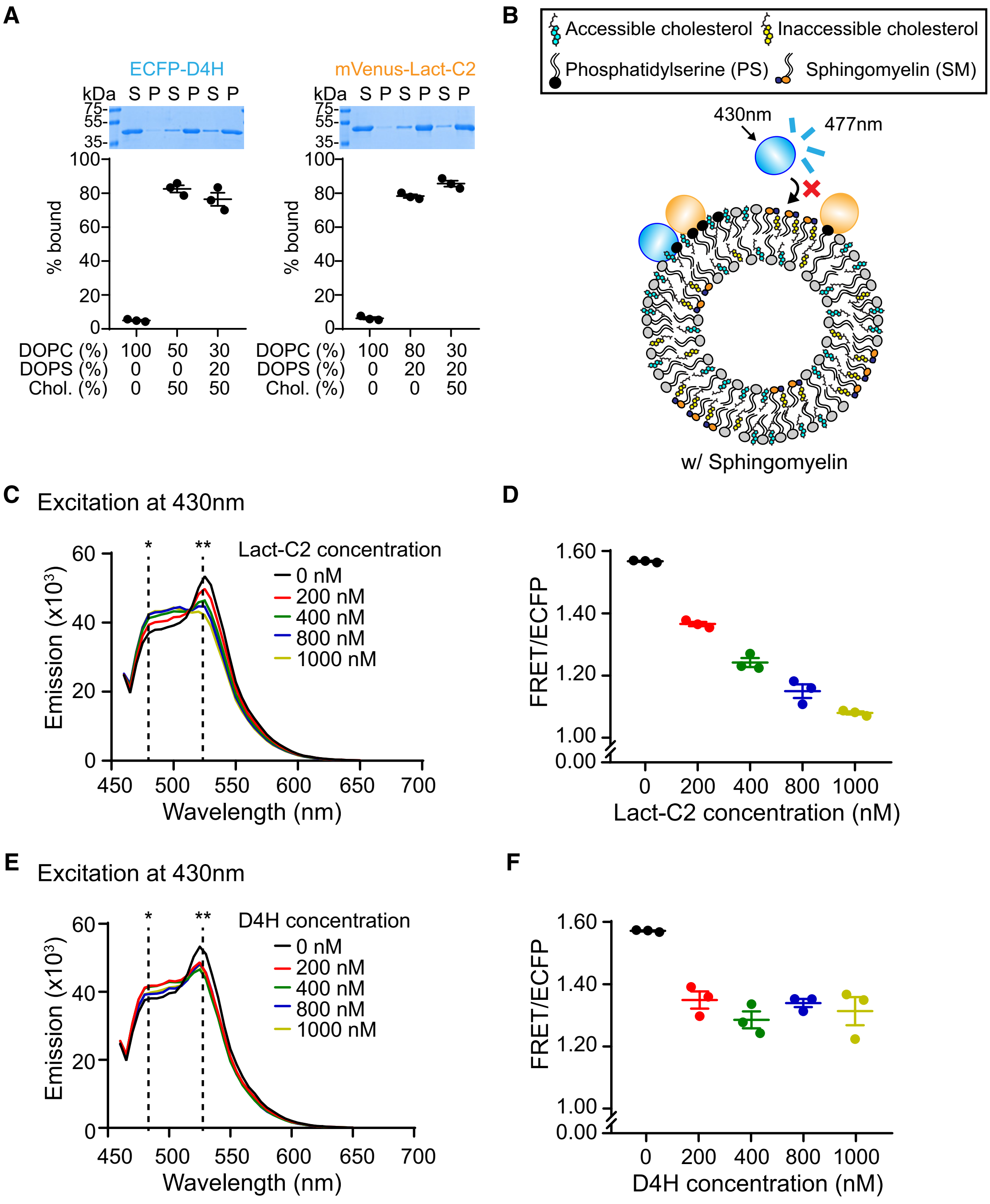

Figure EV1.

◀

Figure EV1. The GRAM domain of GRAMD1b detects the codistribution of accessible cholesterol and PS, which is regulated by sphingomyelin.

A Liposome sedimentation assays of purified ECFP-D4H proteins (accessible cholesterol biosensor) and purified mVenus-Lact-C2 proteins (phosphatidylserine biosensor). Liposomes containing the indicated mole% lipids were incubated with purified proteins as shown. Bound proteins [pellet, (P)] were separated from the unbound proteins [supernatant, (S)], run on SDS–PAGE and visualized by colloidal blue staining (mean ± SEM, $n = 3$ independent experiments). DOPC, phosphatidylcholine (1,2-dioleoyl-sn-glycero-3-phosphocholine); DOPS, phosphatidylserine (1,2-dioleoyl-sn-glycero-3-phospho-L-serine); Chol., cholesterol. Note that the presence of PS does not affect the binding of ECFP-D4H to cholesterol-containing liposomes and that the presence of cholesterol does not affect the binding of mVenus-Lact-C2 to PS containing liposomes.

B Schematic of the *in vitro* FRET assay in the presence of sphingomyelin. Liposomes containing PS, cholesterol, and sphingomyelin were mixed with an accessible cholesterol biosensor (ECFP-D4H) and a PS biosensor (mVenus-Lact-C2). ECFP-D4H was excited at 430 nm and emission from mVenus-Lact-C2 due to FRET between ECFP and mVenus was recorded at 525 nm. The presence of sphingomyelin contributes to sequestration of cholesterol into inaccessible pool, resulting in reduced binding of ECFP-D4H to liposomes and decrease in FRET as observed in Fig 1B.

C Representative emission spectra of mixtures containing ECFP-D4H, mVenus-Lact-C2, and liposomes containing cholesterol (50%), phosphatidylserine (DOPS) (20%), and phosphatidylcholine (DOPC) (30%), with or without untagged Lact-C2 proteins. Asterisks indicate positions of emission maximum of ECFP (*477 nm) and FRET (**525 nm). Note the decrease of FRET signal at 525 nm and increase of ECFP emission at 480 nm upon addition of increasing amount of untagged Lact-C2 proteins into the mixture.

D Quantification of the ratio of FRET signal at 525 nm to ECFP emission at 480 nm (FRET/ECFP) (see Materials and Methods) from mixtures as shown in (C) (mean ± SEM, $n = 3$ independent experiments for all conditions).

E Representative emission spectra of mixtures containing ECFP-D4H, mVenus-Lact-C2, and liposomes containing cholesterol (50%), phosphatidylserine (DOPS) (20%), and phosphatidylcholine (DOPC) (30%), with or without untagged D4H proteins. Asterisks indicate positions of emission maximum of ECFP (*477 nm) and FRET (**525 nm). Note the decrease of FRET signal at 525 nm and increase of ECFP emission at 480 nm upon addition of increasing amount of untagged D4H proteins into the mixture.

F Quantification of the ratio of FRET signal at 525 nm to ECFP emission at 480 nm (FRET/ECFP) (see Materials and Methods) from mixtures as shown in (E) (mean ± SEM, $n = 3$ independent experiments for all conditions).

Figure EV2. A basic patch within the GRAM domain of GRAMD1b is critical for anionic lipid recognition. ▶

A Domain organization of GRAMD1 proteins in comparison to yeast homolog, Lam6/Ltc1.

B Sequence alignment and secondary structure prediction of the GRAM domains from selected GRAMD1s and their homologs. ESPript 3.0 was used to align amino acid sequences and annotate the secondary structure information. Secondary structure prediction is based on the crystal structure of Lam6/Ltc1 (PDB: 5YQR). K161 and R189 residues of human GRAMD1b are indicated by red asterisks. White letters on a red background: strict identity; Red letters: similarity in a group; Blue frames: similarity across groups.

C, D Sequence logos showing the graphical representations of sequence similarity between human GRAMD1b and its homologs (see Materials and Methods) of the regions around K161 residue (C) and R189 residue (D) of human GRAMD1b as indicated in (B) ($n = 106$ aligned sequences). Error bars indicate an approximate Bayesian 95% confidence interval. Black: hydrophobic residues; Green: neutral residues; Blue: basic residues; Red: acidic residues. Note the strong conservation of these K161 and R189 across different species.

 © 2021 The Authors

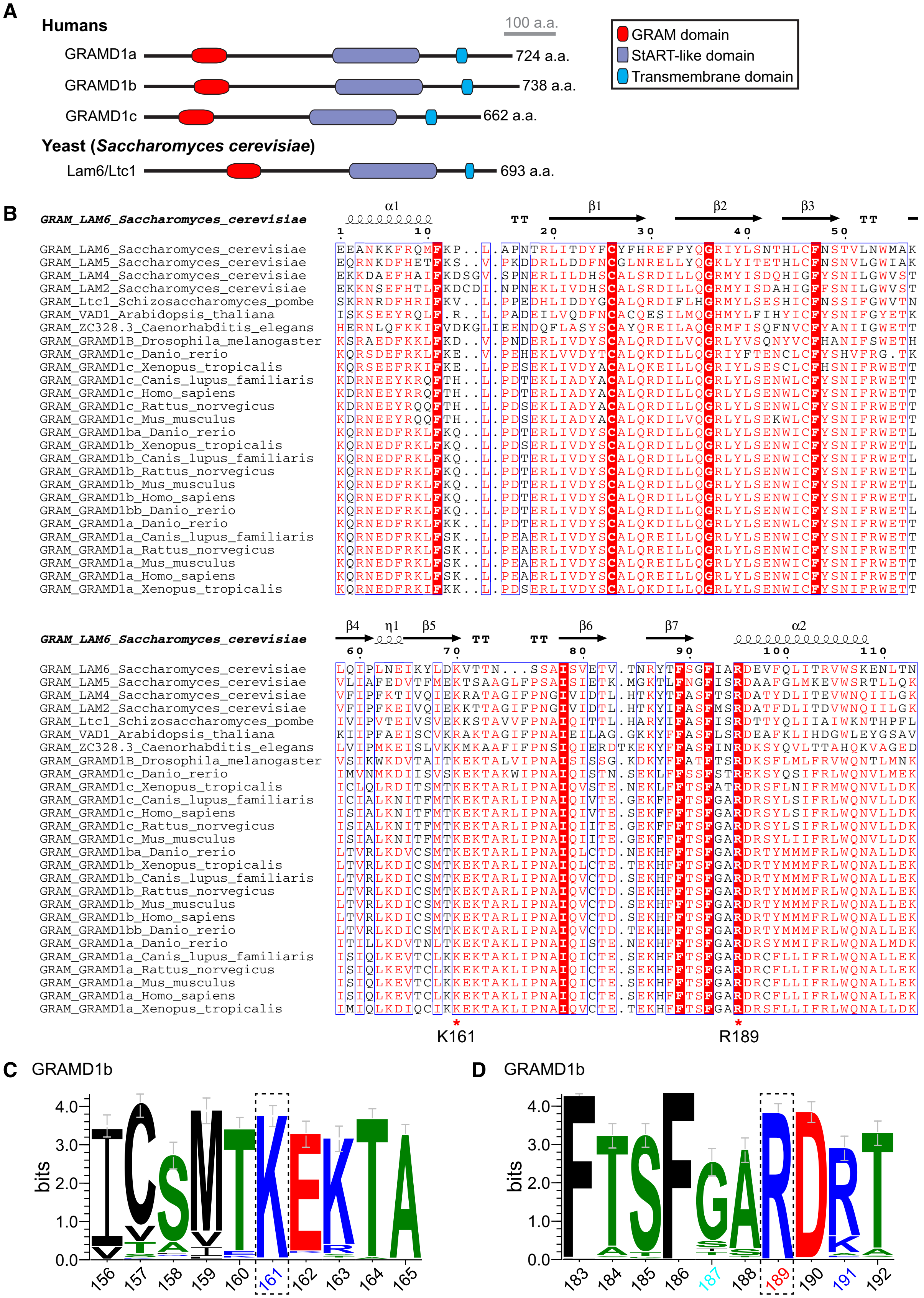
A
Humans
100 a.a.
GRAMD1a
724 a.a.
GRAMD1b
738 a.a.
GRAMD1c
662 a.a.
Yeast (Saccharomyces cerevisiae)
Lam6/Ltc1
693 a.a.
GRAM domain
StART-like domain
Transmembrane domain
B
GRAM_LAM6_Saccharomyces_cerevisiae
GRAM_LAM5_Saccharomyces_cerevisiae
GRAM_LAM4_Saccharomyces_cerevisiae
GRAM_LAM2_Saccharomyces_cerevisiae
GRAM_Ltc1_Schizosaccharomyces_pombe
GRAM_VAD1_Arabidopsis_thaliana
GRAM_ZC328.3_Caenorhabditis_elegans
GRAM_GRAMD1B_Drosophila_melanogaster
GRAM_GRAMD1c_Danio_rerio
GRAM_GRAMD1c_Xenopus_tropicalis
GRAM_GRAMD1c_Canis_lupus_familiaris
GRAM_GRAMD1c_Homo_sapiens
GRAM_GRAMD1c_Rattus_norvegicus
GRAM_GRAMD1c_Mus_musculus
GRAM_GRAMD1ba_Danio_rerio
GRAM_GRAMD1b_Xenopus_tropicalis
GRAM_GRAMD1b_Canis_lupus_familiaris
GRAM_GRAMD1b_Rattus_norvegicus
GRAM_GRAMD1b_Mus_musculus
GRAM_GRAMD1b_Homo_sapiens
GRAM_GRAMD1bb_Danio_rerio
GRAM_GRAMD1a_Danio_rerio
GRAM_GRAMD1a_Canis_lupus_familiaris
GRAM_GRAMD1a_Rattus_norvegicus
GRAM_GRAMD1a_Mus_musculus
GRAM_GRAMD1a_Homo_sapiens
GRAM_GRAMD1a_Xenopus_tropicalis
K161
R189
C GRAMD1b
D GRAMD1b
bits

Figure EV2.

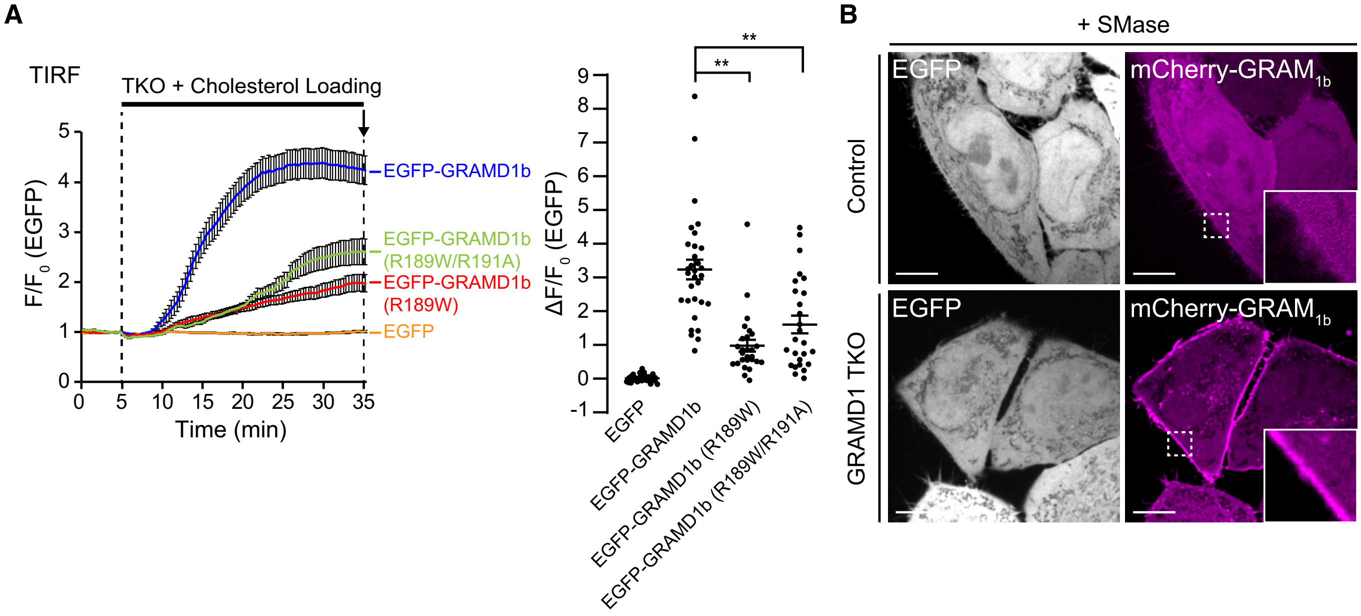

Figure EV3. Cholesterol-sensing property of the GRAM domain is critical for GRAMD1b function.

A Left: Time course of normalized EGFP signal, as assessed by TIRF microscopy, from GRAMD1 TKO (TKO) HeLa cells that stably expressed EGFP or EGFP-tagged GRAMD1b (EGFP-GRAMD1b) constructs as indicated. Cholesterol loading [the treatment with cholesterol/methyl-β-cyclodextrin (MCD) complex (200 μM)] is indicated. Right: Values of $\Delta F/F_0$ corresponding to the end of the experiment as indicated by the arrow [mean ± SEM, $n = 32$ cells (EGFP), $n = 31$ cells (EGFP-GRAMD1b), $n = 27$ cells [EGFP-GRAMD1b (R189W)], $n = 26$ cells [EGFP-GRAMD1b (R189W/R191A)], data are pooled from two independent experiments for each condition; Dunnett's multiple comparisons test, $**P < 0.0001$]. See also Movie EV2.

B Confocal images of live HeLa (control) cells and GRAMD1 TKO cells stably expressing EGFP that were additionally transfected with an accessible PM cholesterol biosensor, mCherry-tagged GRAM domain of GRAMD1b (mCherry-$GRAM_{1b}$). Cells were treated with SMase (100 mU/ml for 1 h at 37°C) before imaging. Insets show at higher magnification the regions indicated by white dashed boxes. Note the very weak PM recruitment of mCherry-$GRAM_{1b}$ in HeLa cells compared to the strong PM recruitment of mCherry-$GRAM_{1b}$ in GRAMD1 TKO cells. Scale bars, 10 μm.

Figure EV4. G187L mutation specifically increases the cholesterol sensitivity of the GRAM domain of GRAMD1b.

A Confocal images of live GRAMD1 TKO HeLa cells expressing mutant versions of EGFP-tagged GRAM domain of GRAMD1b (EGFP-$GRAM_{1b}$) as indicated. Insets show at higher magnification the regions indicated by white dashed boxes. Scale bars, 10 μm.

B Left: Time course of normalized EGFP signal, as assessed by TIRF microscopy, from GRAMD1 TKO (TKO) HeLa cells expressing either wild-type EGFP-$GRAM_{1b}$ (WT) or mutant EGFP-$GRAM_{1b}$ (G187L) as indicated. SMase treatment (100 mU/ml) and methyl-β-cyclodextrin (MCD) treatment (5 mM) are indicated. Note that the fluorescence signal was normalized by the value of 75 min time point. Right: Values of $\Delta F/F_{75}$ corresponding to 70 min time point as indicated by the arrow [mean ± SEM, $n = 41$ cells (WT), $n = 40$ cells (G187L), data are pooled from two independent experiments for each condition; two-tailed unpaired Student's *t*-test, $**P < 0.0001$].

C A confocal image of live GRAMD1 TKO HeLa cells expressing EGFP-$GRAM_{1b}$ (WT). Cells were cultured in the medium supplemented with 10% lipoprotein-deficient serum (LPDS) and mevastatin (50 μM) for 16 h before imaging. An inset shows at higher magnification the region indicated by a white dashed box. Note the absence of PM recruitment. Scale bars, 10 μm.

D–F Liposomes containing the indicated mole% lipids were incubated with purified wild-type $GRAM_{1b}$ (WT) and mutant $GRAM_{1b}$ (G187L) proteins. Bound proteins [pellet, (P)] were separated from the unbound proteins [supernatant, (S)], run on SDS–PAGE and visualized by colloidal blue staining (mean ± SEM, $n = 3$ independent experiments for all the conditions). DPhyPC, 1,2-diphytanoyl-sn-glycero-3-phosphocholine; DOPC, phosphatidylcholine (1,2-dioleoyl-sn-glycero-3-phosphocholine); POPC, 1-palmitoyl-2-oleoyl-glycero-3-phosphocholine; DOPS, phosphatidylserine (1,2-dioleoyl-sn-glycero-3-phospho-L-serine); Chol., cholesterol. Chemical structures of DPhyPC, DOPC, and POPC are shown below (F).

G Both $GRAM_{1b}$ (WT) and $GRAM_{1b}$ (G187L) proteins sense cholesterol accessibility. The mean values of liposome binding efficiency of purified $GRAM_{1b}$ (WT) and $GRAM_{1b}$ (G187L) proteins as assessed by liposome sedimentation assays in (F) and Fig 6H were presented as a heat map.

© 2021 The Authors

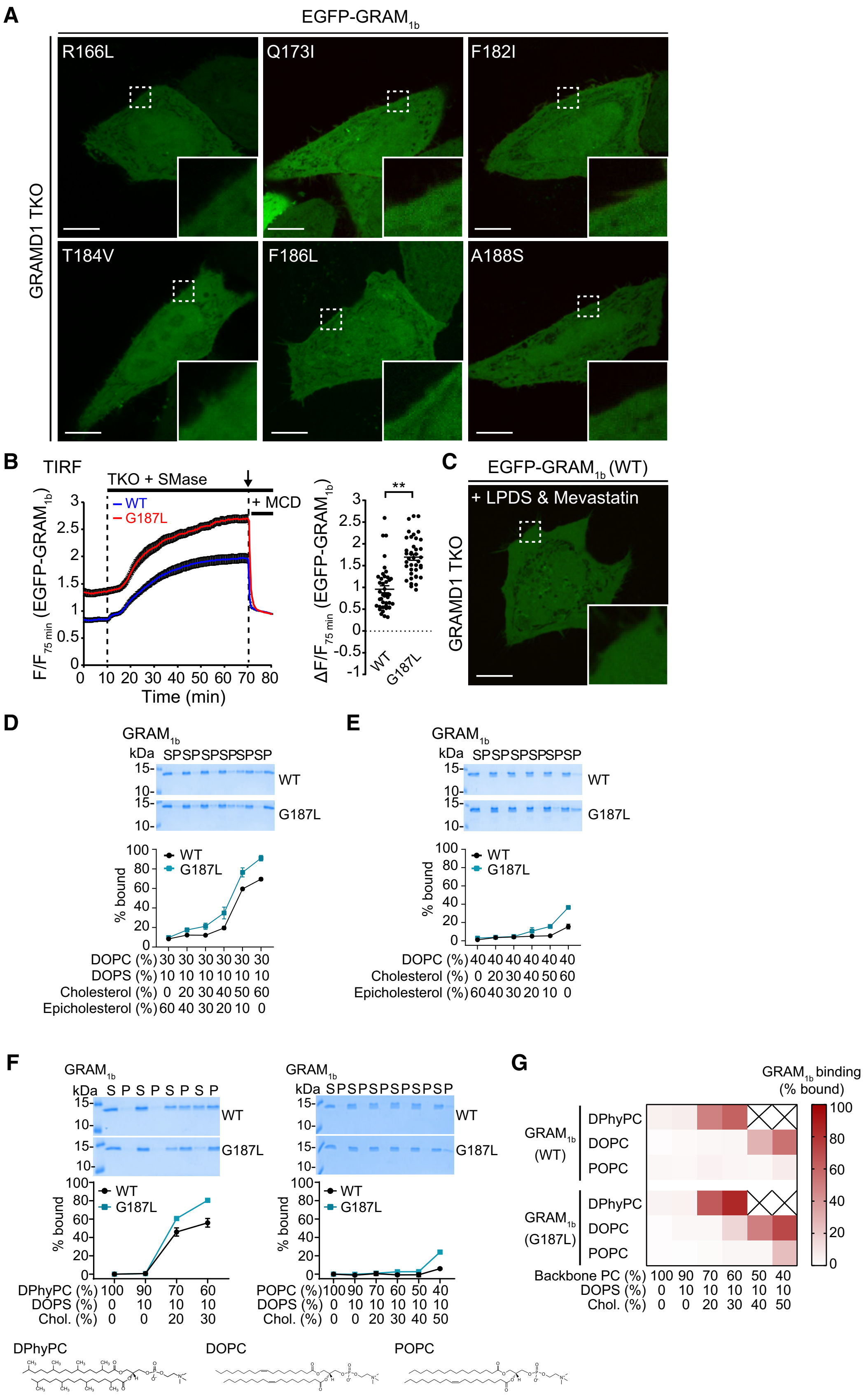

Figure EV4.

© 2021 The Authors

Figure EV5. G187 is critical for determining the sensitivity of the GRAM domain to accessible PM cholesterol and regulating GRAMD1b-dependent cholesterol transport.

A Effects of mutation of G187 on the property of the GRAM domain of GRAMD1b ($GRAM_{1b}$) to sense transient expansions of the accessible pool of PM cholesterol. Quantification of the ratio of PM signals to the cytosolic signals of wild-type EGFP-tagged $GRAM_{1b}$ (EGFP-$GRAM_{1b}$) (WT) and mutant versions of EGFP-$GRAM_{1b}$, as assessed by confocal microscopy and line scan analysis from GRAMD1 TKO HeLa cells, expressing indicated constructs, with or without SMase treatment (100 mU/ml for 1 h at 37°C) or with SMase treatment followed by 5 min treatment with Methyl-β-cyclodextrin (MCD) (5 mM) (mean ± SEM, *n* = 20 cells from two independent experiments for untreated and SMase-treated conditions; *n* = 10 cells from one experiment for SMase & MCD-treated conditions). Amino acids are ranked according to Goldman, Engelman and Steitz (GES) hydrophobicity scale with the most hydrophobic amino acid on the left. Red dashed lines indicate the average values of WT with or without SMase treatment.

B A representative size exclusion chromatography (SEC) profile of purified mutant GRAMD1b proteins (G187L). The peak fraction was subjected to SDS–PAGE followed by colloidal blue staining.

C Representative time course of DHE transfer from L_{PM} to L_{ER} mediated by GRAMD1b–G187L. Purified GRAMD1b–G187L proteins (0.05 μM, 0.1 μM or 0.2 μM) were added at time 0. Addition of buffer alone (0 μM) was used as a control.

D Concentration dependency of GRAMD1b-dependent DHE transfer from L_{PM} to L_{ER}. Note the concentration-dependent DHE transfer mediated by GRAMD1b–WT and GRAMD1b–G187L proteins. Note also the absence of DHE transfer by GRAMD1b-R189W/R191A proteins at any concentration tested (mean ± SEM, *n* = 3 independent experiments for all the conditions).

E Liposome sedimentation assays of purified wild-type (WT) and mutant (G187L) GRAMD1b proteins. Proteins were incubated with sucrose-loaded L_{ER}. Bound proteins [pellet, (P)] were separated from the unbound proteins [supernatant, (S)], run on SDS–PAGE and visualized by colloidal blue staining (mean ± SEM, *n* = 3 independent experiments for all conditions).

F Lysates of wild-type (control) and GRAMD1 TKO HeLa cells that stably expressed either EGFP or EGFP-tagged GRAMD1b (EGFP-GRAMD1b) constructs as indicated [wild-type (WT), G187L mutant (G187L)] were processed by SDS–PAGE and immunoblotted (IB) with anti-GFP, anti-GRAMD1b, and anti-actin antibodies.

G Left: Time course of normalized EGFP signal, as assessed by TIRF microscopy, from GRAMD1 TKO (TKO) HeLa cells that stably expressed EGFP or EGFP-GRAMD1b constructs as indicated. Cholesterol loading [the treatment with cholesterol/MCD complex (200 μM)] is indicated. Right: Values of $\Delta F/F_0$ corresponding to the end of the experiment as indicated by the arrow [mean ± SEM, *n* = 27 cells (EGFP), *n* = 23 cells (EGFP-GRAMD1b), *n* = 26 cells [EGFP-GRAMD1b (G187L)], data are pooled from two independent experiments for each condition; Tukey's multiple comparisons test, ***P* < 0.0001]. See also Movie EV4.

H Amphotericin B resistance of SMase-treated GRAMD1 TKO (TKO) HeLa cells that stably expressed either EGFP or EGFP-GRAMD1b constructs as indicated [wild-type (WT) or G187L mutant (G187L)]. Left: Cells that had been pre-treated with SMase (100 mU/ml) for 3 h at 37°C were treated with indicated concentration of Amphotericin B for 20 min at 37°C. After overnight recovery in culture media, cell viability was measured by detecting ATP present in each well via luminescence (see Materials and Methods). The same number of cells were seeded in each well before SMase treatment. Note the strong resistance against Amphotericin B of GRAMD1 TKO HeLa cells that stably expressed EGFP-GRAMD1b–G187L. Right: Quantification of cell viability with increasing amount of Amphotericin B. Note the resistance of GRAMD1 TKO cells that stably expressed EGFP-GRAMD1b–G187L compared to GRAMD1 TKO cells that stably expressed either EGFP or EGFP-GRAMD1b–WT (mean ± SEM, *n* = 3 independent experiments for each condition).

 © 2021 The Authors

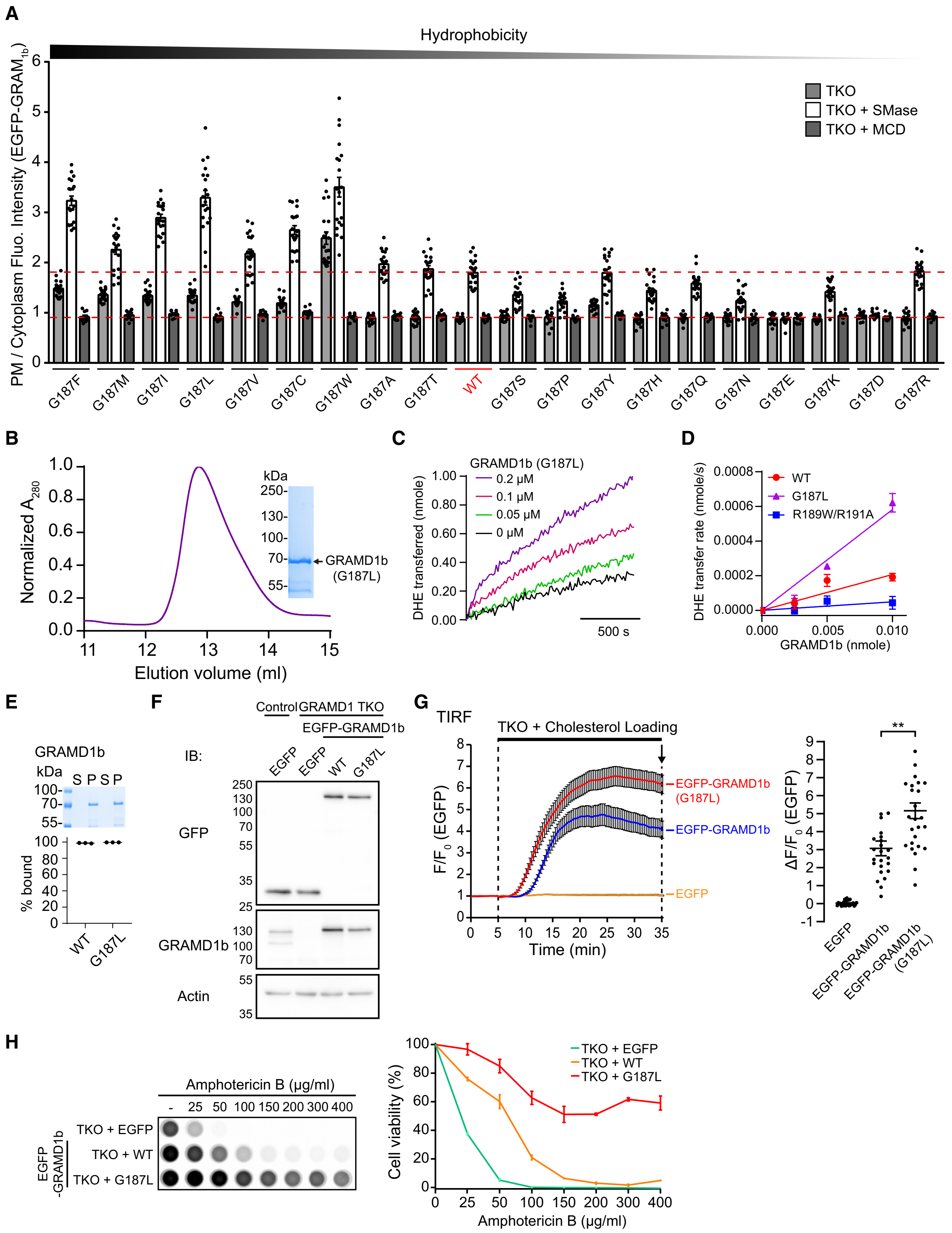
A
Hydrophobicity
PM / Cytoplasm Fluo. Intensity (EGFP-GRAM1b)
TKO
TKO + SMase
TKO + MCD
G187F G187M G187I G187L G187V G187C G187W G187A G187T WT G187S G187P G187Y G187H G187Q G187N G187E G187K G187D G187R
B
Normalized A280
Elution volume (ml)
kDa
GRAMD1b (G187L)
C
GRAMD1b (G187L)
0.2 μM
0.1 μM
0.05 μM
0 μM
DHE transferred (nmole)
500 s
D
WT
G187L
R189W/R191A
DHE transfer rate (nmole/s)
GRAMD1b (nmole)
E
GRAMD1b
kDa
S P S P
% bound
WT G187L
F
Control GRAMD1 TKO
EGFP-GRAMD1b
IB:
EGFP EGFP WT G187L
GFP
GRAMD1b
Actin
G
TIRF
TKO + Cholesterol Loading
F/F0 (EGFP)
Time (min)
EGFP-GRAMD1b (G187L)
EGFP-GRAMD1b
EGFP
ΔF/F0 (EGFP)
**
EGFP EGFP-GRAMD1b EGFP-GRAMD1b (G187L)
H
Amphotericin B (μg/ml)
- 25 50 100 150 200 300 400
TKO + EGFP
TKO + WT
TKO + G187L
EGFP -GRAMD1b
Cell viability (%)
Amphotericin B (μg/ml)
TKO + EGFP
TKO + WT
TKO + G187L

Figure EV5.
