# Supplementary material for: Molecular basis of accessible plasma membrane cholesterol recognition by the GRAM domain of GRAMD1b
Source: EMBO J. 2021 Feb 19;40(6):e106524. doi: 10.15252/embj.2020106524 (PMC7957428; doi:10.15252/embj.2020106524)
Supplement: Supplementary file 3 — Table EV1 [file EMBJ-40-e106524-s004.docx]

**TABLE EV1**

| **REAGENT or RESOURCE** | **SOURCE** | **IDENTIFIER** |
| --- | --- | --- |
| **Antibodies** | | |
| Anti-GFP | Abcam | RRID:AB_303395 |
| Anti-GRAMD1b | Proteintech | 24905-1-AP |
| Anti-Actin | EMD Millipore | RRID:AB_2223041 |
| Anti-SREBP-2 | Santa Cruz Biotechnology | RRID: [AB_2194250](http://antibodyregistry.org/AB_2194250) |
| Goat Anti-Rabbit IgG (H+L)-HRP Conjugate | Bio Rad | RRID:AB_11125142 |
| Goat Anti-Mouse IgG (H+L)-HRP Conjugate | Bio Rad | RRID:AB_11125547 |
| **Bacterial Strains** | | |
| E. coli BL21-DE3 Rosetta | NTU PPP |  |
| NEB® Turbo Competent E. coli | NEB | C2984I |
| **Chemicals, Peptides, and Recombinant Proteins** | | |
| 1,2-dioleoyl-sn-glycero-3-phosphocholine (DOPC) | Avanti Polar Lipids | 850375 |
| 1,2-dioleoyl-sn-glycero-3-phospho-L-serine (sodium salt) (DOPS) | Avanti Polar Lipids | 840035 |
| N-oleoyl-D-erythro-sphingosylphosphorylcholine (18:1 SM) | Avanti Polar Lipids | 860587 |
| 1-palmitoyl-2-oleoyl-glycero-3-phosphocholine (POPC) | Avanti Polar Lipids | 850457 |
| 1,2-diphytanoyl-sn-glycero-3-phosphocholine (DPhyPC) | Avanti Polar Lipids | 850356 |
| 1-palmitoyl-2-oleoyl-sn-glycero-3-phosphoethanolamine (POPE) | Avanti Polar Lipids | 850757 |
| 1,2-dioleoyl-sn-glycero-3-phosphate (sodium salt) (18:1 PA) | Avanti Polar Lipids | 840875C |
| L-α-phosphatidylinositol (Liver, Bovine) (sodium salt) (Liver PI) | Avanti Polar Lipids | 840042 |
| L-α-phosphatidylinositol-4-phosphate (Brain, Porcine) (ammonium Salt) (Brain PI(4)P) | Avanti Polar Lipids | 840045 |
| L-α-phosphatidylinositol-4,5-bisphosphate (Brain, Porcine) (ammonium salt) (Brain PI(4,5)P_2_) | Avanti Polar Lipids | 840046 |
| Dehydroergosterol (DHE) | Avanti Polar Lipids | 810253 |
| 18:1 DGS-NTA(Ni) | Avanti Polar Lipids | 790404 |
| 1,2-dioleoyl-sn-glycero-3-phosphoethanolamine-N-(5-dimethylamino-1-naphthalenesulfonyl) (ammonium salt) (18:1 Dansyl PE) | Avanti Polar Lipids | 810330 |
| Desmosterol | Avanti Polar Lipids | 700060 |
| Cholesterol | Sigma-Aldrich | C8667 |
| Epicholesterol | Steraloids | C6730-000 |
| Cholesteryl acetate | Steraloids | C6761-000 |
| Lysozyme from chicken egg white | Sigma-Aldrich | 62970 |
| DNase | Sigma-Aldrich | DN25 |
| Tris (2-carboxyethyl) phosphine (TCEP) | Sigma-Aldrich | C4706 |
| Isopropyl-1-thio-B-d-galactopyranoside (IPTG) | ThermoFisher | R0392 |
| cOmplete™, EDTA-free Protease Inhibitor Cocktail | Merck | 11873580001 |
| COLLOIDAL BLUE STAINING KIT | Thermofisher | LC6025 |
| HisPur™ Ni-NTA Resin | Thermofisher | 88222 |
| Benzonase® Nuclease | Santa Cruz Biotechonology | sc-202391 |
| Sphingomyelinase from Bacillus cereus (SMase) | Sigma-Aldrich/Merck | S9396 |
| Puromycin | Stemcell | 73342 |
| Q5 High-Fidelity DNA Polymerase | NEB | M0491S |
| Opti-MEM™ I Reduced Serum Medium | Thermo scientific | 31985070 |
| Lipofectamine 2000 Reagent | Invitrogen | 11668-019 |
| Fetal Bovine Serum | Gibco | 10270-106 |
| DMEM(4.5g/l Glucose) with L-Gln, without Sodium Pyruvate | Nacalai Tesque, Japan | 08459-35 |
| Amphotericin B | Sigma-Aldrich/Merck | A4888 |
| **Critical Commercial Assays** | | |
| Q5 Site-Directed Mutagenesis Kit | NEB | E0552S |
| NEBuilder HiFi DNA Assembly Cloning Kit | NEB | E5520S |
| BCA Protein Assay Kit | NEB | 23225 |
| CellTiter-Glo® Luminescent Cell Viability Assay Kit. | Promega | G7570 |
| **Experimental Models: Cell Lines** | | |
| HeLa M | Others | Gift from Pietro De Camilli |
| HeLa GRAMD1 TKO (labeled as GRAMD1 TKO) |  | Naito T. (2019) |
| HeLa expressing EGFP | This paper |  |
| GRAMD1 TKO expressing EGFP | This paper |  |
| GRAMD1 TKO expressing GRAMD1b | This paper |  |
| GRAMD1 TKO expressing GRAMD1b R189W | This paper |  |
| GRAMD1 TKO expressing GRAMD1b R189W R191A | This paper |  |
| GRAMD1 TKO expressing GRAMD1b G187L | This paper |  |
| **Oligonucleotides** | | |
| GTTTGGGGCCtggGATAGGACAT | This paper | GRAMD1b_R189W_F |
| GAAGTGAAGAAGTGCTTTTCTGAATCAG | This paper | GRAMD1b_R189W_R |
| tgccACATATATGATGATGTTCCGGC | This paper | GRAMD1b_R189W R191A_F |
| tcccaGGCCCCAAACGAAGTGAA | This paper | GRAMD1b_R189W R191A_R |
| TTCCATGACTgctGAAAAAACAGCTCG | This paper | GRAM1b_K161A_F |
| CAGATGTCTTTCAAACGG | This paper | GRAM1b_K161A_R |
| CACTTCGTTTctcGCCCGGGATAG | This paper | GRAMD1b_G187L_F |
| AAGAAGTGCTTTTCTGAATC | This paper | GRAMD1b_G187L_R |
| GGCCCGGGATgccACATATATGATG | This paper | GRAMD1b_R191A_F |
| CCAAACGAAGTGAAGAAG | This paper | GRAMD1b_R191A_R |
| AAAAACAGCTgctCTCATTCCCAATGC | This paper | GRAMD1b_R166L_F |
| TCTTTAGTCATGGAACAG | This paper | GRAMD1b_R166L_R |
| CAATGCCATCattGTTTGCACTGATTCAGAAAAG | This paper | GRAMD1b_Q173I_F |
| GGAATGAGGCGAGCTGTT | This paper | GRAMD1b_Q173I_R |
| AGAAAAGCACatcTTCACTTCGTTTG | This paper | GRAMD1b_F182I_F |
| GAATCAGTGCAAACTTGG | This paper | GRAMD1b_F182I_R |
| GCACTTCTTCgtcTCGTTTGGGGC | This paper | GRAMD1b_T184V_F |
| TTTTCTGAATCAGTGCAAAC | This paper | GRAMD1b_T184V_R |
| CTTCACTTCGctcGGGGCCCGGG | This paper | GRAMD1b_F186L_F |
| AAGTGCTTTTCTGAATCAGTGCAAACTTG | This paper | GRAMD1b_F186L_R |
| TTCGTTTGGGtcgCGGGATAGGAC | This paper | GRAMD1b_A188S_F2 |
| GTGAAGAAGTGCTTTTCTG | This paper | GRAMD1b_A188S_R |
| CACTTCGTTTaagGCCCGGGATA | This paper | GRAMD1b_G187K_F |
| AAGAAGTGCTTTTCTGAATCAG | This paper | GRAMD1b_G187K_R |
| CACTTCGTTTtggGCCCGGGATA | This paper | GRAMD1b_G187W_F |
| AAGAAGTGCTTTTCTGAATCAGTGC | This paper | GRAMD1b_G187W_R |
| CACTTCGTTTcggGCCCGGGATA | This paper | GRAMD1b_G187R_F |
| AAGAAGTGCTTTTCTGAATCAGTGC | This paper | GRAMD1b_G187R_R |
| CACTTCGTTTcacGCCCGGGATAG | This paper | GRAMD1b_G187H_F |
| AAGAAGTGCTTTTCTGAATC | This paper | GRAMD1b_G187H_R |
| CACTTCGTTTgacGCCCGGGATAG | This paper | GRAMD1b_G187D_F |
| AAGAAGTGCTTTTCTGAATCAG | This paper | GRAMD1b_G187D_R |
| CACTTCGTTTgaaGCCCGGGATAG | This paper | GRAMD1b_G187E_F |
| AAGAAGTGCTTTTCTGAATCAG | This paper | GRAMD1b_G187E_R |
| CACTTCGTTTtccGCCCGGGATAG | This paper | GRAMD1b_G187S_F |
| AAGAAGTGCTTTTCTGAATC | This paper | GRAMD1b_G187S_R |
| CACTTCGTTTacaGCCCGGGATAG | This paper | GRAMD1b_G187T_F |
| AAGAAGTGCTTTTCTGAATC | This paper | GRAMD1b_G187T_R |
| CACTTCGTTTaatGCCCGGGATAG | This paper | GRAMD1b_G187N_F |
| AAGAAGTGCTTTTCTGAATC | This paper | GRAMD1b_G187N_R |
| CACTTCGTTTcaaGCCCGGGATAG | This paper | GRAMD1b_G187Q_F |
| AAGAAGTGCTTTTCTGAATC | This paper | GRAMD1b_G187Q_R |
| CACTTCGTTTtgcGCCCGGGATAG | This paper | GRAMD1b_G187C_F |
| AAGAAGTGCTTTTCTGAATC | This paper | GRAMD1b_G187C_R |
| CACTTCGTTTcccGCCCGGGATAG | This paper | GRAMD1b_G187P_F |
| AAGAAGTGCTTTTCTGAATC | This paper | GRAMD1b_G187P_R |
| CACTTCGTTTgctGCCCGGGATAG | This paper | GRAMD1b_G187A_F |
| AAGAAGTGCTTTTCTGAATC | This paper | GRAMD1b_G187A_R |
| CACTTCGTTTgttGCCCGGGATAG | This paper | GRAMD1b_G187V_F |
| AAGAAGTGCTTTTCTGAATC | This paper | GRAMD1b_G187V_R |
| CACTTCGTTTatcGCCCGGGATAG | This paper | GRAMD1b_G187I_F |
| AAGAAGTGCTTTTCTGAATC | This paper | GRAMD1b_G187I_R |
| CACTTCGTTTatgGCCCGGGATAG | This paper | GRAMD1b_G187M_F |
| AAGAAGTGCTTTTCTGAATCAG | This paper | GRAMD1b_G187M_R |
| CACTTCGTTTtttGCCCGGGATAG | This paper | GRAMD1b_G187F_F |
| AAGAAGTGCTTTTCTGAATC | This paper | GRAMD1b_G187F_R |
| CACTTCGTTTtacGCCCGGGATAG | This paper | GRAMD1b_G187Y_F |
| AAGAAGTGCTTTTCTGAATC | This paper | GRAMD1b_G187Y_R |
| ggatGCCacatatatgatgatgttccg | This paper | GRAMD1b_G187L R191A_F |
| cgggcGAGaaacgaagtgaagaagtg | This paper | GRAMD1b_G187L R191A_R |
| ACTTTAAGAAGGAGATATACCATGGGAAGTTGGTATAATGTGTTAAGCC | This paper | 5'NcoI C-GRAMD1b (82-529) |
| AGTGGTGGTGGTGGTGGTGCTCGAGTCTGTGCATCTCAGCCAA | This paper | 3'XhoI C-GRAMD1b (82-548) |
| CTTCCAATCCATGGCCGGAAAGGGAAAAATAAACTTAGAT | This paper | 5'NcoI_pNIC28_D4H |
| GCTCGAATTCGGATCCTCAATTGTAAGTAATACTAGATCC | This paper | 3'BamHI_pNIC28_D4H |
| GCCATATGGCTAGCTGCACAGAACCGCTTGGGCTGAAGGA | This paper | 5'NheI_C2 |
| GTGGTGCTCGAGCTAACAACCAAGTAATTCAACGCGAA | This paper | 3'XhoI_EGFP-C2 |
| GTACTTCCAATCCATGGTCTCCAAGGGTGAAGAGCTTTTC | This paper | 5'NcoI_ECFP-D4H |
| ATTCGGATCCTCAATTGTAAGTAATACTAGATCCAGGGTA | This paper | 3'BamHI_ECFP-D4H |
| TGTACTTCCAATCCATGGTAAGTAAAGGTGAGGAACTGTT | This paper | 5'NcoI_mVenus-Lact-C2 |
| ACGGAGCTCGAATTCGGATCCCTAACAGCCCAGCAGCTCCACTCG | This paper | 3'BamHI_pNIC28_mCherry-Lact-C2 |
| ctgtacttccaatccATGGTCTCCAAGGGTGAAGAGCTTTTCACCGGGGTGGTCCCTATTCTTGTTGAACTTGACGGAGACGTAAATGGGCACAAATTTTCCGTAAGCGGTGAAGGTGAGGGGGATGCCACCTACGGTAAATTGACATTGAAATTTATCTGCACGACGGGTAAGTTACCCGTCCCCTGGCCCACTTTAGTCACAACTCTGACTTGGGGCGTTCAGTGTTTCAGCCGTTATCCTGATCACATGAAGCAGCATGATTTCTTCAAGTCTGCGATGCCGGAGGGTTACGTCCAAGAGCGCACAATCTTTTTCAAAGACGACGGGAACTATAAGACACGTGCCGAAGTGAAATTTGAGGGAGATACCCTGGTAAATCGTATTGAACTGAAAGGAATTGACTTCAAAGAGGACGGCAACATCTTAGGGCATAAGTTGGAATATAATTACATTTCGCATAATGTGTATATCACAGCCGACAAGCAGAAAAATGGGATCAAGGCGAATTTTAAAATTCGCCATAACATTGAAGACGGAAGCGTTCAGTTAGCAGATCATTATCAGCAAAATACTCCGATCGGCGACGGTCCGGTGTTACTTCCCGATAACCACTACCTTTCGACCCAATCTGCGCTTTCGAAAGACCCGAACGAGAAGCGTGATCATATGGTGTTACTGGAGTTCGTCACTGCGGCGGGAATCACGCTGGGGATGGATGAACTTTATAAGtccggactcagatctGGAAAGGGAAAAATAAACTTAGATCATAGTGGAGCCTATGTTGCACAGTTTGAAGTAGCCTGGGATGAAGTTTCATATGACAAAGAAGGAAATGAAGTTTTAACTCATAAAACATGGGATGGAAATTATCAAtcaAAAACAGCTCACTATTCAACAGTAATACCTCTTGAAGCTAATGCAAGAAATATAAGAATAAAAGCAAGAGAGTGTACAGGCCTTGCTTGGGAATGGTGGAGAGATGTTATAAGTGAATATGATGTTCCATTAACAAATAATATAAATGTTTCAATATGGGGAACAACTTTATACCCTGGATCTAGTATTACTTACAATTGAGGATCCGAATTCGA |  | ECFP-D4H |
| tgtacttccaatccATGGTAAGTAAAGGTGAGGAACTGTTCACGGGAGTAGTTCCAATTTTGGTTGAGCTGGATGGGGACGTAAACGGACATAAATTCAGCGTAAGCGGGGAAGGTGAGGGAGATGCTACATATGGAAAACTGACACTGAAATTGATTTGTACCACAGGCAAATTACCTGTCCCGTGGCCTACCTTGGTGACCACTCTGGGGTACGGGCTTCAATGCTTTGCGCGTTACCCCGACCACATGAAGCAACACGACTTCTTTAAGTCAGCGATGCCGGAAGGCTATGTCCAAGAACGCACTATCTTTTTCAAGGATGATGGGAACTATAAGACTCGCGCGGAAGTGAAGTTCGAGGGTGATACTTTAGTTAATCGTATCGAACTGAAGGGAATCGACTTCAAAGAAGACGGCAATATTCTTGGGCACAAGTTGGAATACAATTATAACAGTCACAACGTGTACATCACGGCGGACAAGCAAAAGAATGGTATCAAAGCTAATTTCAAAATTCGTCACAATATTGAAGACGGAGGGGTGCAGCTTGCGGACCATTACCAACAAAATACCCCAATCGGCGACGGACCGGTGTTACTGCCAGACAATCATTACTTGAGTTATCAAAGTAAGCTGAGTAAAGACCCTAATGAGAAGCGTGACCATATGGTATTGTTGGAGTTCGTAACAGCCGCGGGTATCACGTTGGGTATGGATGAACTTTACAAGtccggactcagatcttgcactgaacccctaggcctgaaggataataccatccccaacaagcagatcacagcctccagctactacaaaacctggggcctgagtgcctttagctggtttccctactacgcacgactggataatcagggcaagttcaacgcctggaccgcccagaccaacagtgcctctgagtggctgcagattgacctgggctcccagaagcgagtcacgggcatcatcacccagggtgcccgagactttggccacattcaatatgtggctgcctacagggtggcctatggtgatgatggtgtgacctggactgagtacaaggacccgggggcctcagaaagcaagattttccctggtaacatggacaataattcccacaagaagaacatatttgagacgccgttccaggctcgcttcgtgcggatccagcccgtggcctggcacaaccgtatcaccctgcgagtggagctgctgggctgttagGGATCCGAATTCGA |  | mVenus-Lact-C2 |
| GGCAGCCATATGGCTAGCGTTAGTAAAGGTGAGGAACTGTTCACAGGGGTGGTCCCCATTCTTGTGGAGTTAGATGGAGATGTGAACGGACATAAATTCAGCGTAAGTGGCGAGGGCGAGGGCGACGCCACCTACGGAAAATTGACCCTTAAATTCATTTGCACTACTGGAAAATTGCCAGTCCCGTGGCCTACATTGGTCACCACCCTGACTTACGGAGTGCAGTGCTTCTCTCGCTACCCTGACCACATGAAACAACACGACTTCTTTAAGTCCGCTATGCCTGAGGGCTACGTGCAGGAACGCACGATTTTTTTCAAAGATGACGGCAATTACAAGACCCGCGCAGAAGTTAAATTTGAGGGCGACACACTGGTAAATCGCATCGAATTAAAGGGAATTGACTTTAAAGAGGACGGAAACATTCTTGGTCATAAACTGGAGTATAATTACAATAGTCACAATGTATATATCATGGCCGACAAACAGAAAAATGGGATTAAGGTTAATTTTAAAATTCGTCATAACATTGAAGACGGCTCTGTGCAATTAGCAGACCATTACCAGCAAAATACACCGATCGGAGACGGCCCTGTTTTACTTCCTGATAACCATTACTTATCCACGCAGAGTGCCCTTAGTAAGGACCCAAACGAGAAACGTGATCATATGGTATTACTGGAGTTCGTCACGGCTGCTGGCATTACGCTTGGTATGGATGAATTATATAAGGGTGGAGGCGGTTCCGGCGGAGGTGGCTCCGGCGGTGGCGGATCCTGCACAGAACCGCTTGGGCTGAAGGACAACACGATTCCGAACAAACAGATTACGGCGTCGTCTTATTACAAAACATGGGGTCTTTCCGCTTTTTCGTGGTTCCCATACTATGCGCGTTTAGATAATCAAGGTAAGTTCAATGCATGGACTGCGCAGACTAACTCCGCGTCGGAATGGTTACAAATTGACTTGGGGAGCCAGAAACGTGTTACGGGTATTATCACACAAGGTGCCCGCGATTTCGGACACATTCAATACGTCGCTGCGTATCGCGTCGCTTACGGAGATGATGGCGTTACTTGGACGGAGTACAAAGACCCCGGGGCGAGCGAGAGCAAAATCTTTCCCGGTAACATGGATAACAACAGCCATAAGAAGAACATCTTCGAGACGCCGTTCCAAGCTCGCTTCGTTCGCATTCAGCCAGTCGCTTGGCATAATCGTATTACACTTCGCGTTGAATTACTTGGTTGTtagCTCGAGCACCAC |  | EGFP-Lact-C2 |
| gagaacctgtacttccaatccatggtgagcaagggcgag | This paper | D4H_F |
| gcttcctttcgggctttgtcaattgttaattgtaggttatggaactacctgg | This paper | D4H_R |
| **Recombinant DNA** | | |
| pNIC28-Bsa4 His-GRAMD1b_92-207_ (GRAM_1b_) | This paper | BE36 |
| pNIC28-Bsa4 His-GRAM_1b_ (R189W) | This paper | BE47 |
| pNIC28-Bsa4 His-GRAM_1b_ (R189W&R191A) | This paper | BE98 |
| pNIC28-Bsa4 His-GRAM_1b_ (G187L) | This paper | BE99 |
| pNIC28-Bsa4 His-GRAM_1b_ (K161A) | This paper | BE121 |
| pNIC28-Bsa4 His-GRAM_1b_ (R191A) | This paper | BE117 |
| pET28b(+) GRAMD1b_82-548_-His | This paper | BE19 |
| pET28b(+) GRAMD1b_82-548_-His (R189W) | This paper | BE34 |
| pET28b(+) GRAMD1b_82-548_-His (R189W&R191A) | This paper | BE81 |
| pET28b(+) GRAMD1b_82-548_-His (G187L) | This paper | BE101 |
| pNIC28-Bsa4 His-ECFP-D4H | This paper | BE82 |
| pNIC28-Bsa4 His-mVenus-Lact-C2 | This paper | BE83 |
| pNIC28-Bsa4 His-D4H | This paper | BE75 |
| pET28b(+) His-Lact-C2 | This paper | BE28 |
| pNIC28-Bsa4 EGFP-D4H | Naito, T. (2019) | BE66 |
| EGFP-GRAM_1b_ | Naito, T. (2019) | D2 |
| EGFP-GRAM_1b_ (R189W) | This paper | TN78 |
| EGFP-GRAM_1b_ (R191A) | This paper | D61 |
| EGFP-GRAM_1b_ (R189W/R191A) | This paper | TN100 |
| EGFP-GRAM_1b_ (K161A) | This paper | D94 |
| EGFP-GRAM_1b_ (K161A/R191A) | This paper | D96 |
| EGFP-GRAM_1b_ (R166L) | This paper | BE92 |
| EGFP-GRAM_1b_ (Q173I) | This paper | BE86 |
| EGFP-GRAM_1b_ (F182I) | This paper | BE89 |
| EGFP-GRAM_1b_ (T184V) | This paper | BE94 |
| EGFP-GRAM_1b_ (F186L) | This paper | BE90 |
| EGFP-GRAM_1b_ (A188S) | This paper | BE95 |
| EGFP-GRAM_1b_ (G187L) | This paper | BE84 |
| EGFP-GRAM_1b_ (G187K) | This paper | D59 |
| EGFP-GRAM_1b_ (G187W) | This paper | D60 |
| EGFP-GRAM_1b_ (G187R) | This paper | D76 |
| EGFP-GRAM_1b_ (G187H) | This paper | D77 |
| EGFP-GRAM_1b_ (G187D) | This paper | D78 |
| EGFP-GRAM_1b_ (G187E) | This paper | D79 |
| EGFP-GRAM_1b_ (G187S) | This paper | D80 |
| EGFP-GRAM_1b_ (G187T) | This paper | D81 |
| EGFP-GRAM_1b_ (G187N) | This paper | D82 |
| EGFP-GRAM_1b_ (G187Q) | This paper | D83 |
| EGFP-GRAM_1b_ (G187C) | This paper | D84 |
| EGFP-GRAM_1b_ (G187P) | This paper | D85 |
| EGFP-GRAM_1b_ (G187A) | This paper | D86 |
| EGFP-GRAM_1b_ (G187V) | This paper | D87 |
| EGFP-GRAM_1b_ (G187I) | This paper | D88 |
| EGFP-GRAM_1b_ (G187M) | This paper | D89 |
| EGFP-GRAM_1b_ (G187F) | This paper | D90 |
| EGFP-GRAM_1b_ (G187Y) | This paper | D91 |
| EGFP-GRAM_1b_ (G187L/R191A) | This paper | D125 |
| mCherry-GRAM_1b_ | This paper | TN199 |
| pNIC28-Bsa4 mCherry-D4H | This paper | D148 |
| pLJM1-EGFP | Addgene | #19319 |
| pLJM1-EGFP-GRAMD1b WT | This paper | D49 |
| pLJM1-EGFP-GRAMD1b (R189W) | This paper | D52 |
| pLJM1-EGFP-GRAMD1b (RWRA) | This paper | D53 |
| pLJM1-EGFP-GRAMD1b (G187L) | This paper | D51 |
| mCherry-LactC2 | Other | Gift from Pietro De Camilli |
| pMD2.G | Addgene | #12259 |
| pRSV-REV | Addgene | #12253 |
| pMDL/pRRE | Addgene | #12251 |
| **Software and Algorithms** | | |
| GraphPad |  |  |
| PyMoL | The PyMOL Molecular Graphics System, Version 1.2r3pre, Schrödinger, LLC. |  |
| ImageJ | Schneider et al., 2012 | https://imagej.nih.gov/ij/ |
| **Other** | | |
| 100 nm NanoSizer Extruder | T&T Scientific | TT-002-0001 |
| **Liposomes used in lipid transfer assay** | | |
| L_ER_: (15% DGS-NTA (Ni), 20% 1-palmitoyl-2-oleoyl-sn-glycero-3-phosphoethanolamine (POPE), 65% DOPC) | Saheki Lab | This study |
| L_PM_: (10% DHE, 30% cholesterol, 10% DOPS, 2.5% Dansyl-PE (DNS-PE), 47.5% DOPC) | Saheki Lab | This study |
