# Supplementary material for: Molecular basis of accessible plasma membrane cholesterol recognition by the GRAM domain of GRAMD1b
Source: EMBO J. 2021 Feb 19;40(6):e106524. doi: 10.15252/embj.2020106524 (PMC7957428; doi:10.15252/embj.2020106524)
Supplement: Supplementary file 5 — Movie EV2 [file EMBJ-40-e106524-s013.zip › MovieEV2_Legends.docx]

**Movie EV2. Cholesterol- and PS- sensing properties of the GRAM domain are critical for GRAMD1b recruitment to the PM upon cholesterol loading**

GRAMD1 TKO HeLa cells that stably expressed EGFP-GRAMD1b constructs as indicated were imaged under TIRF microscopy. Images were taken every 20 seconds, and 200 μM cholesterol/MCD were added at 5 min time point. Image size, 55.1 µm x 55.1 µm.
