# Supplementary figures and images for: Molecular basis of accessible plasma membrane cholesterol recognition by the GRAM domain of GRAMD1b
Source: EMBO J. 2021 Feb 19;40(6):e106524. doi: 10.15252/embj.2020106524 (PMC7957428; doi:10.15252/embj.2020106524)

Figure EV5B

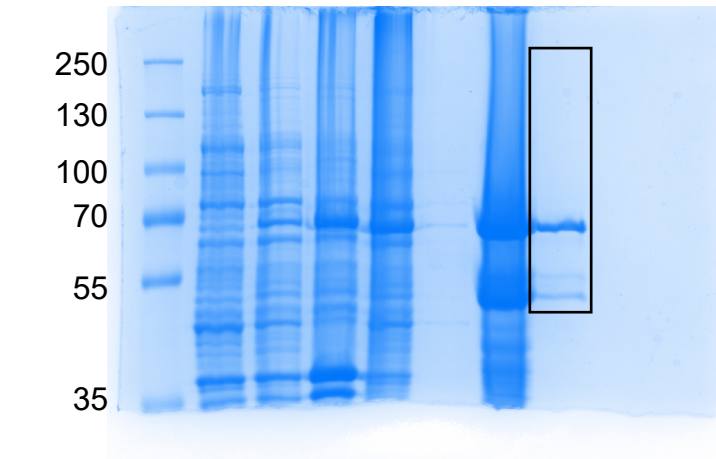

Figure EV5E

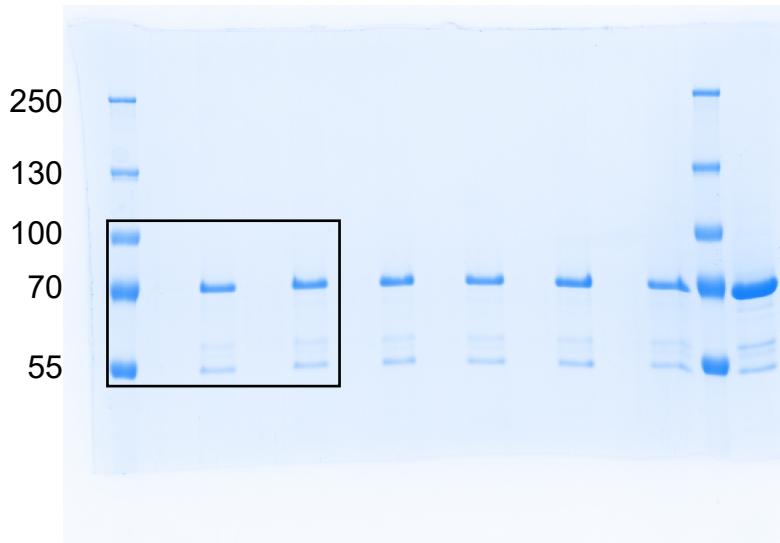

Figure EV5F

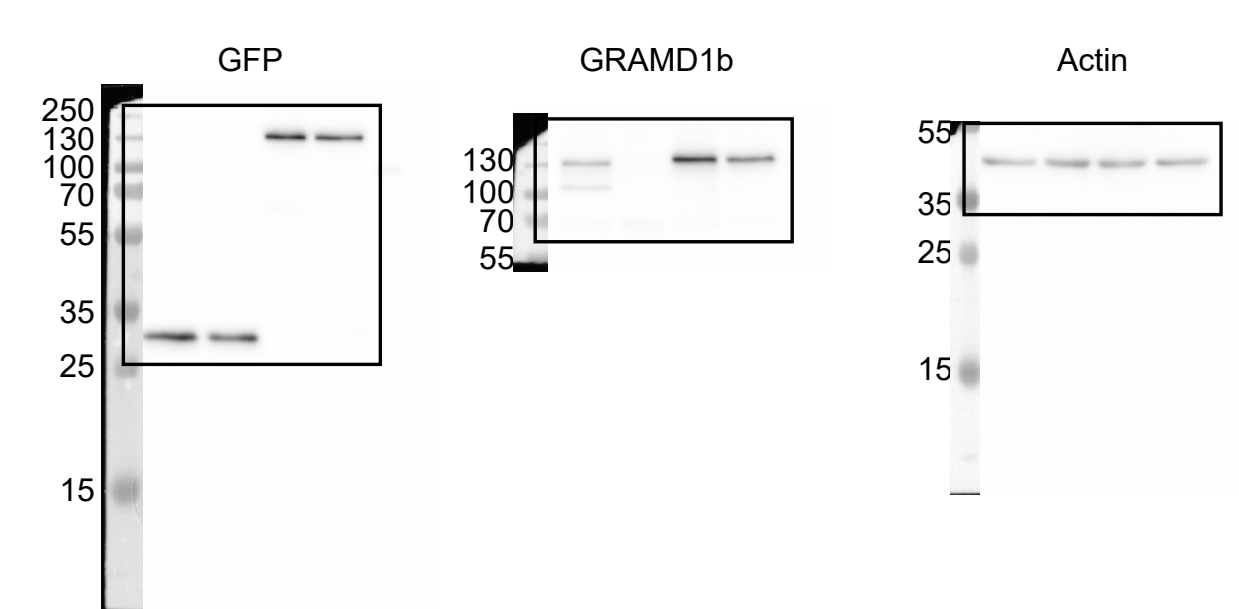

Supplement: Supplementary file 8 — Source Data for Expanded View and Appendix [file EMBJ-40-e106524-s002.zip › EMBOJ-2020-106524_SourceDataforFigureEV5.pdf]

**Figure EV1A**

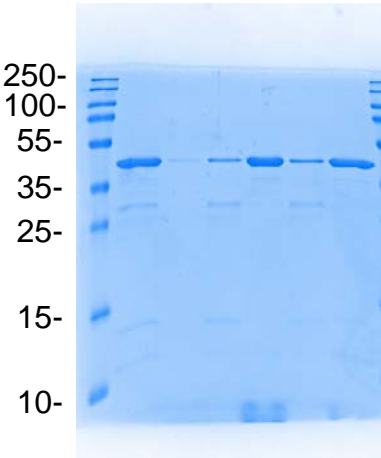

Left image: ECFP-D4H

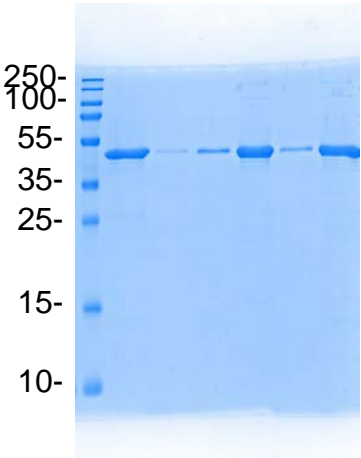

Right image: mVenus-Lact-C2

Supplement: Supplementary file 8 — Source Data for Expanded View and Appendix [file EMBJ-40-e106524-s002.zip › EMBOJ-2020-106524_SourceDataforFigureEV1.pdf]

**Figure Appendix S2A**

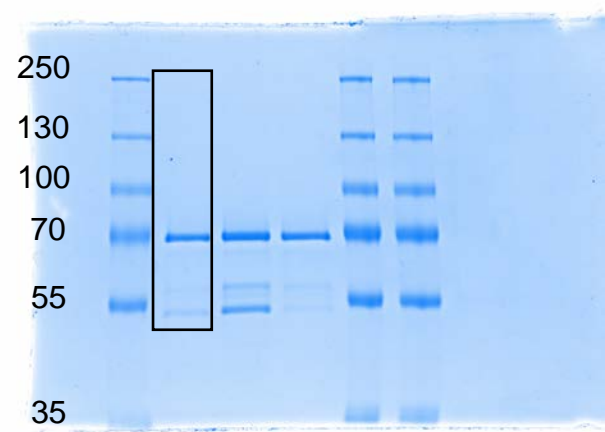

**Figure Appendix S2B**

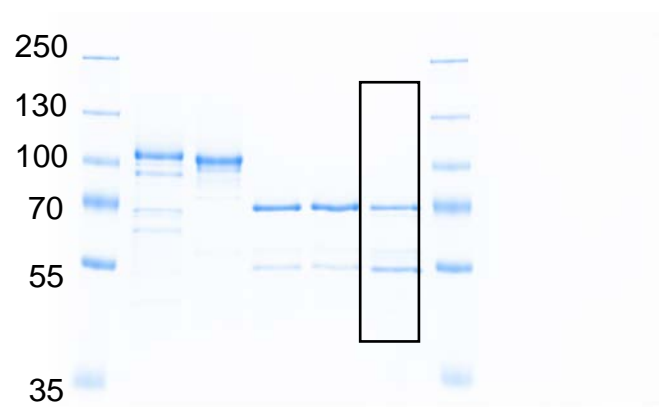

**Figure Appendix S2C**

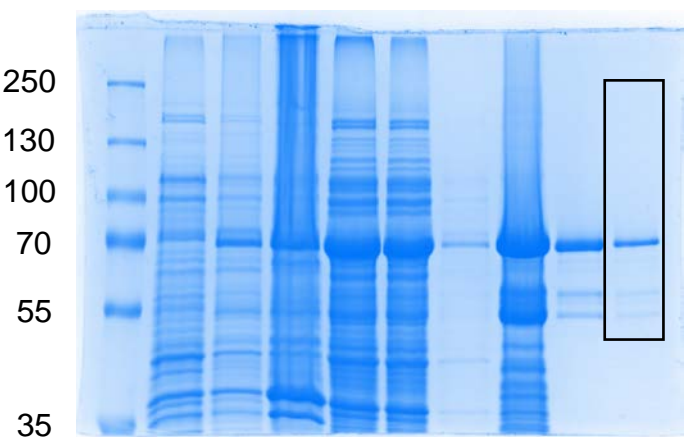

Supplement: Supplementary file 8 — Source Data for Expanded View and Appendix [file EMBJ-40-e106524-s002.zip › EMBOJ-2020-106524_SourceDataforFigureAppendixS2.pdf]

Figure Appendix S1A

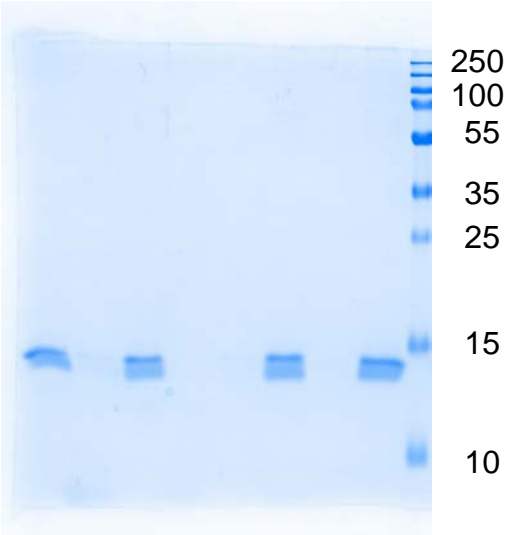

Figure Appendix S1 Source Data

Supplement: Supplementary file 8 — Source Data for Expanded View and Appendix [file EMBJ-40-e106524-s002.zip › EMBOJ-2020-106524_SourceDataforFigureAppendixS1.pdf]

**Figure EV4D**

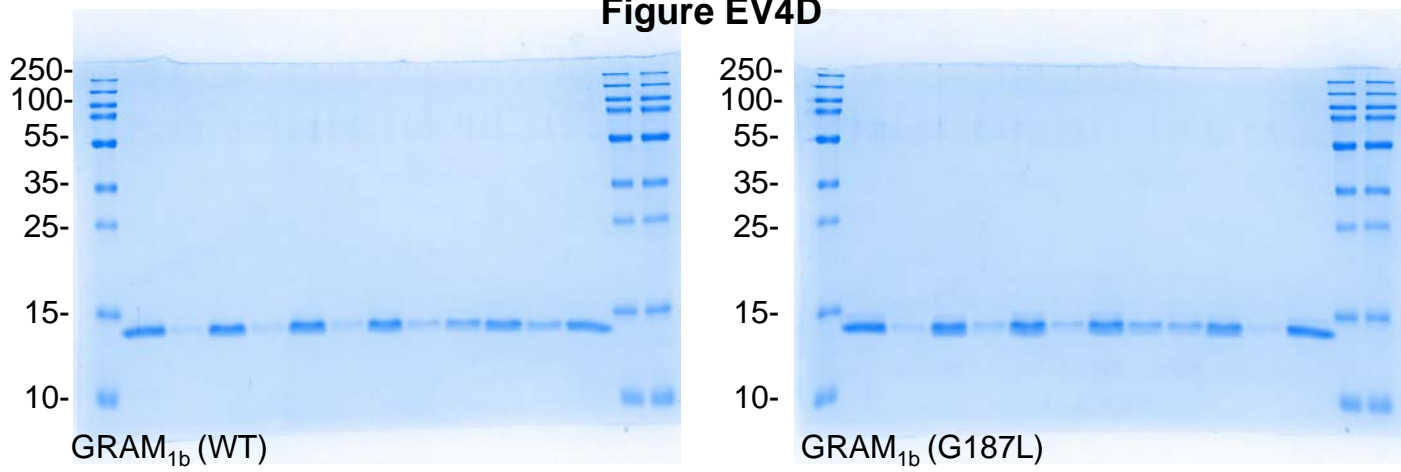

**Figure EV4E**

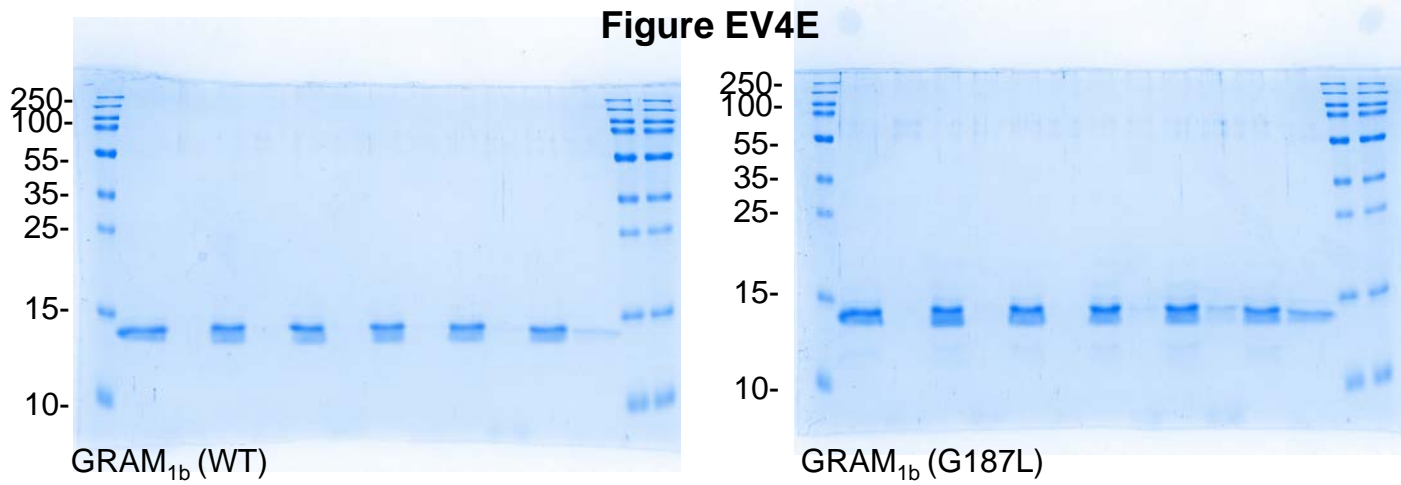

**Figure EV4F**

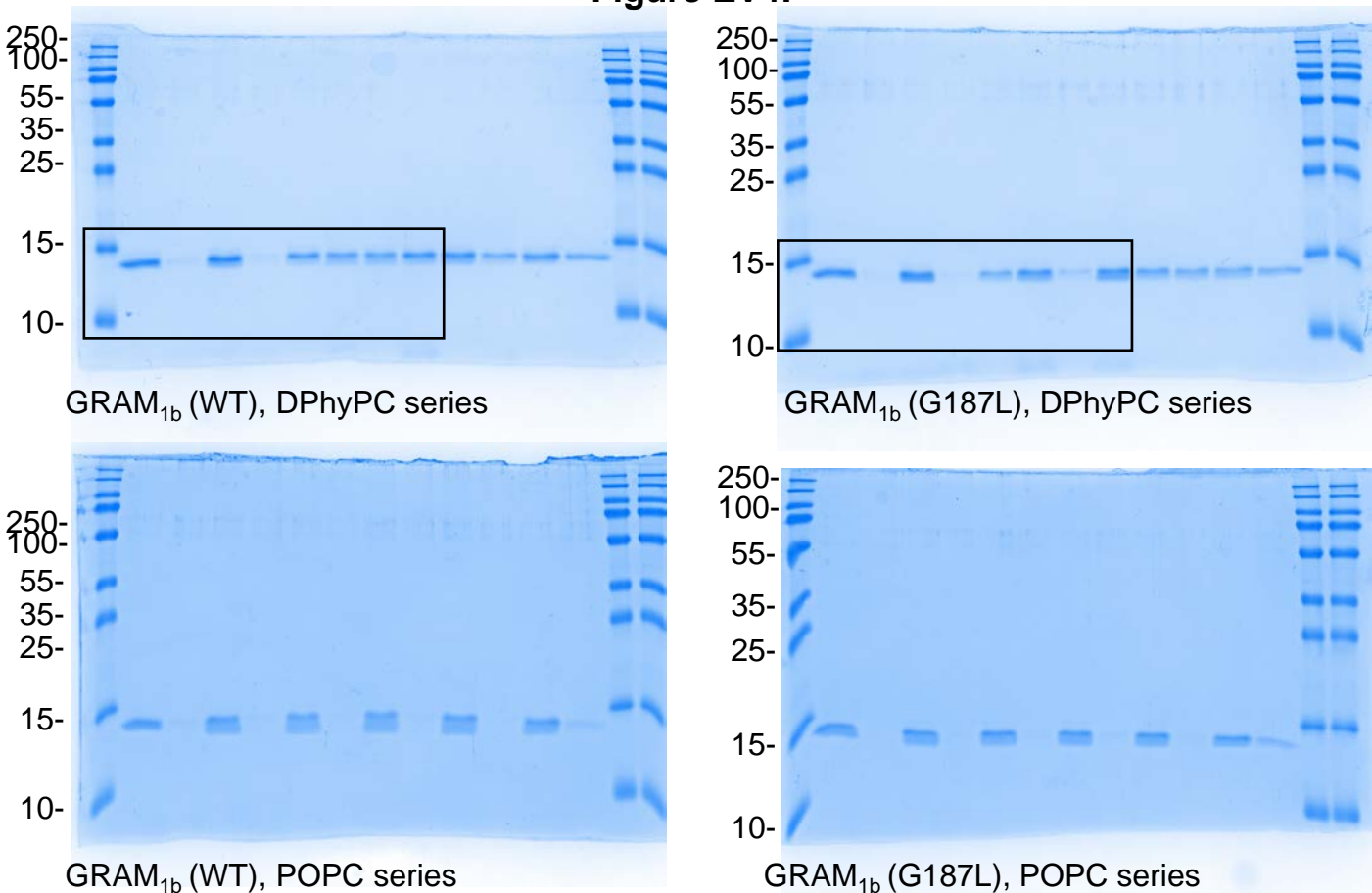

**Figure EV4 Source Data**

Supplement: Supplementary file 8 — Source Data for Expanded View and Appendix [file EMBJ-40-e106524-s002.zip › EMBOJ-2020-106524_SourceDataforFigureEV4.pdf]

**Figure Appendix S3A**

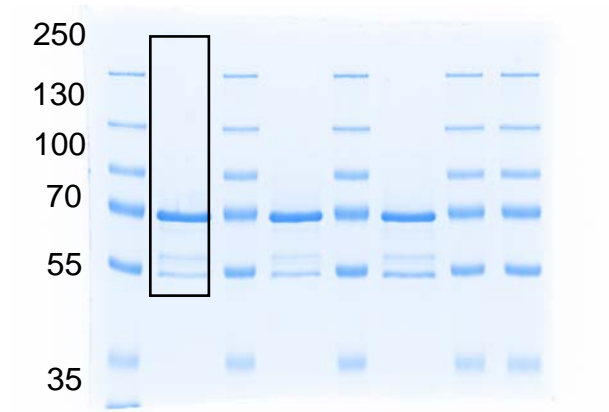

**Figure Appendix S3B**

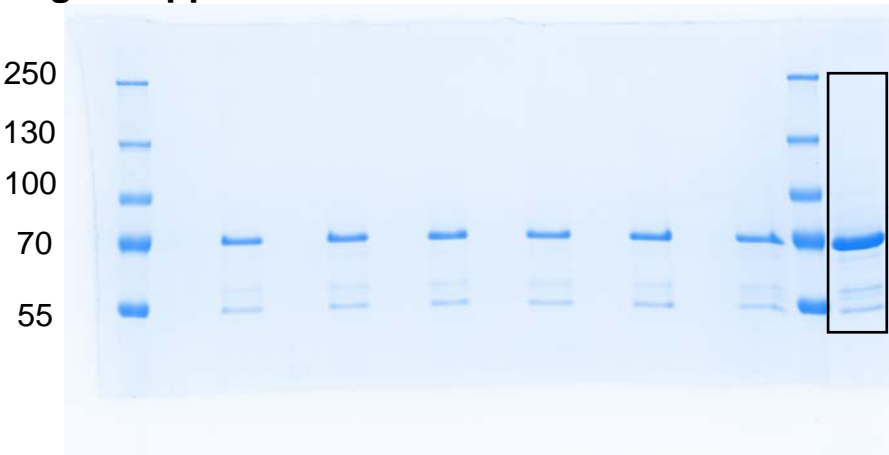

**Figure Appendix S3E**

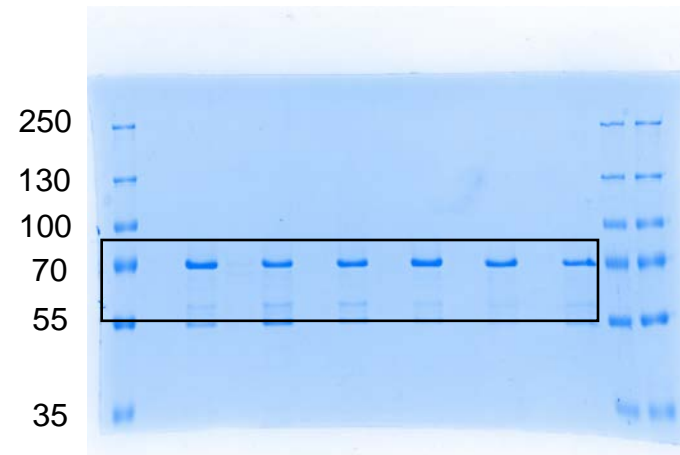

Supplement: Supplementary file 8 — Source Data for Expanded View and Appendix [file EMBJ-40-e106524-s002.zip › EMBOJ-2020-106524_SourceDataforFigureAppendixS3.pdf]

**Figure 3B**

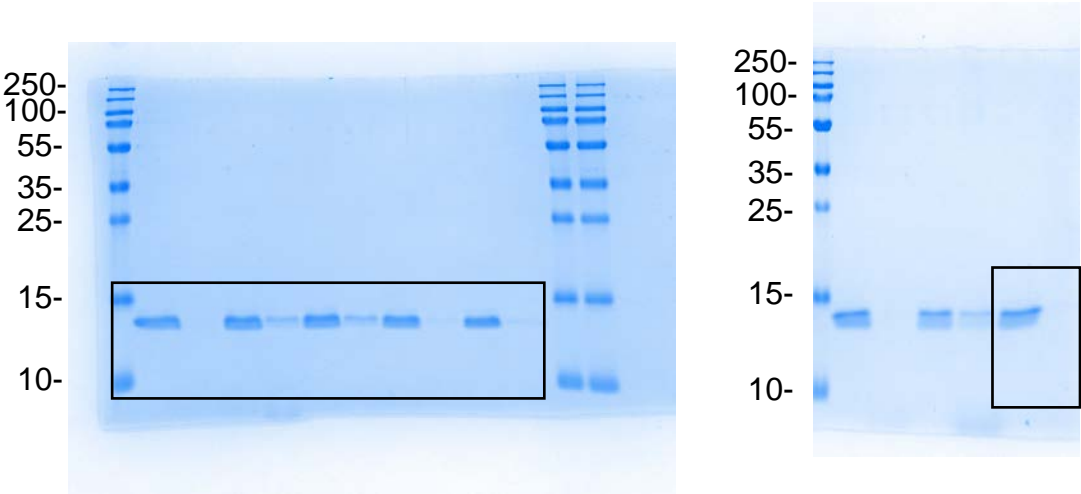

**Figure 3D**

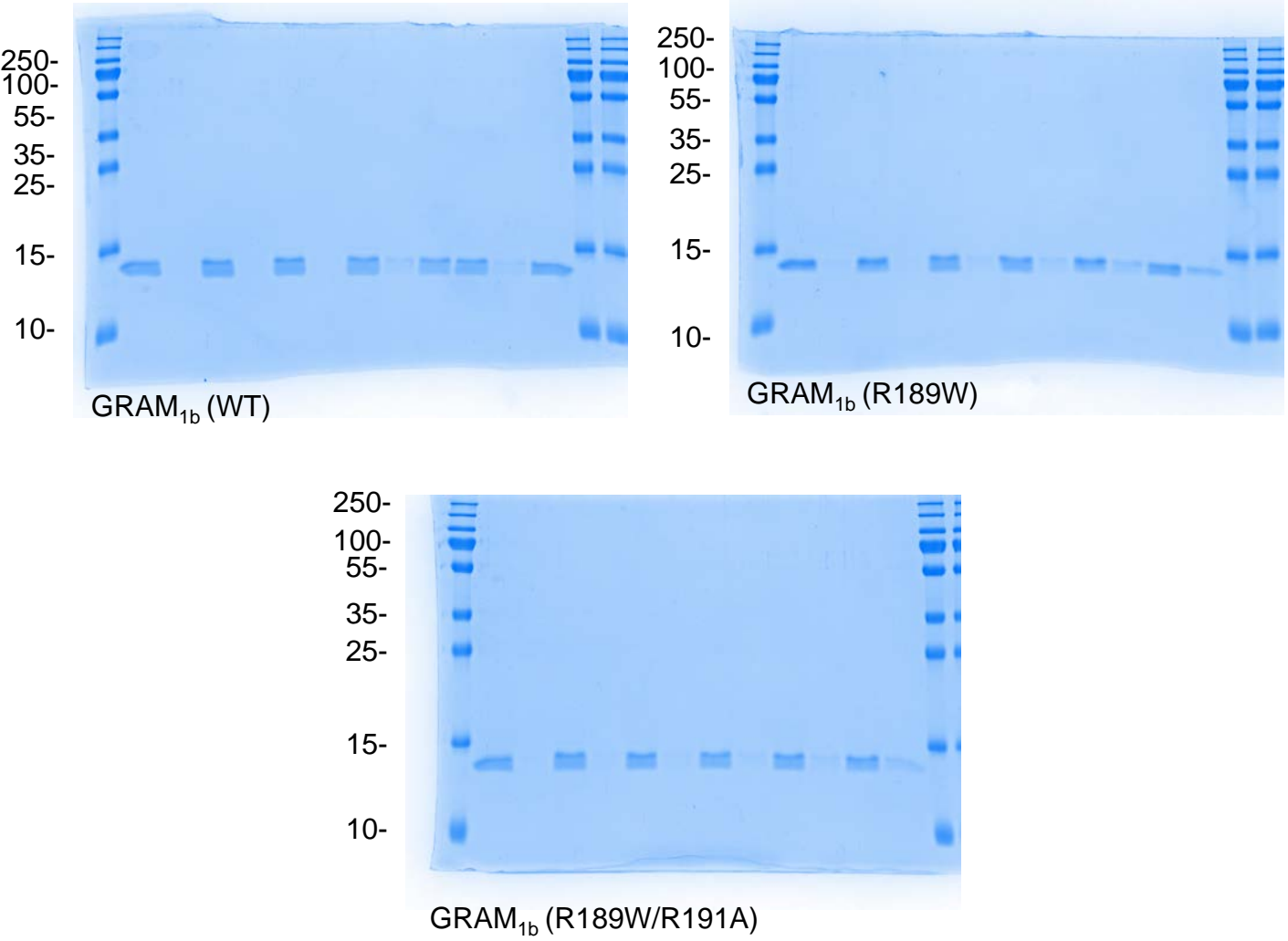

**Figure 3 Source Data**

Figure 3E

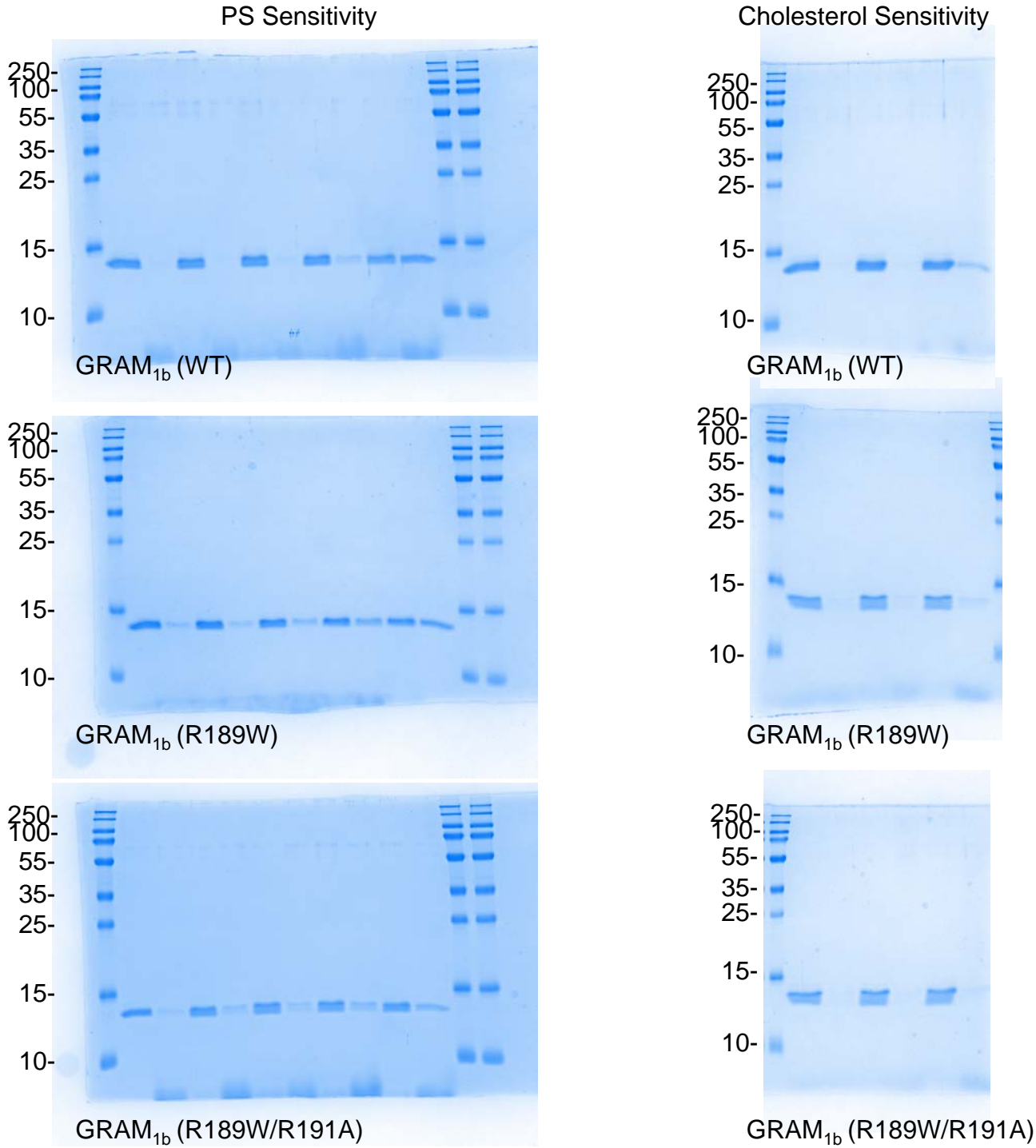

Figure 3 Source Data

Supplement: Supplementary file 11 — Source Data for Figure 3 [file EMBJ-40-e106524-s011.pdf]

**Figure 5A**

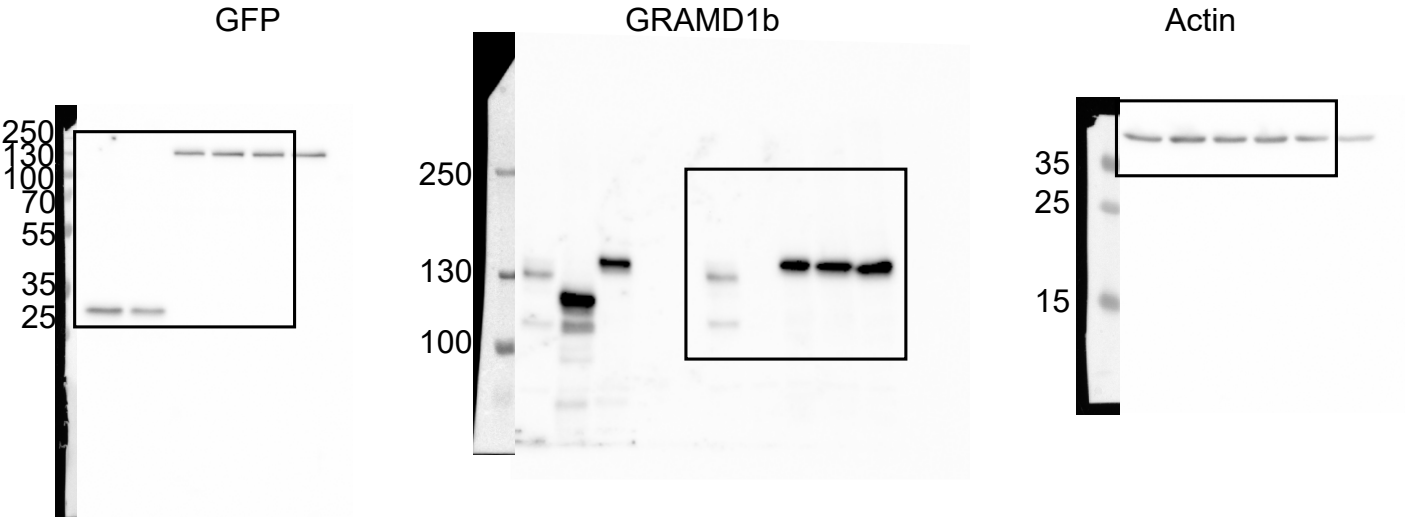

**Figure 5E**

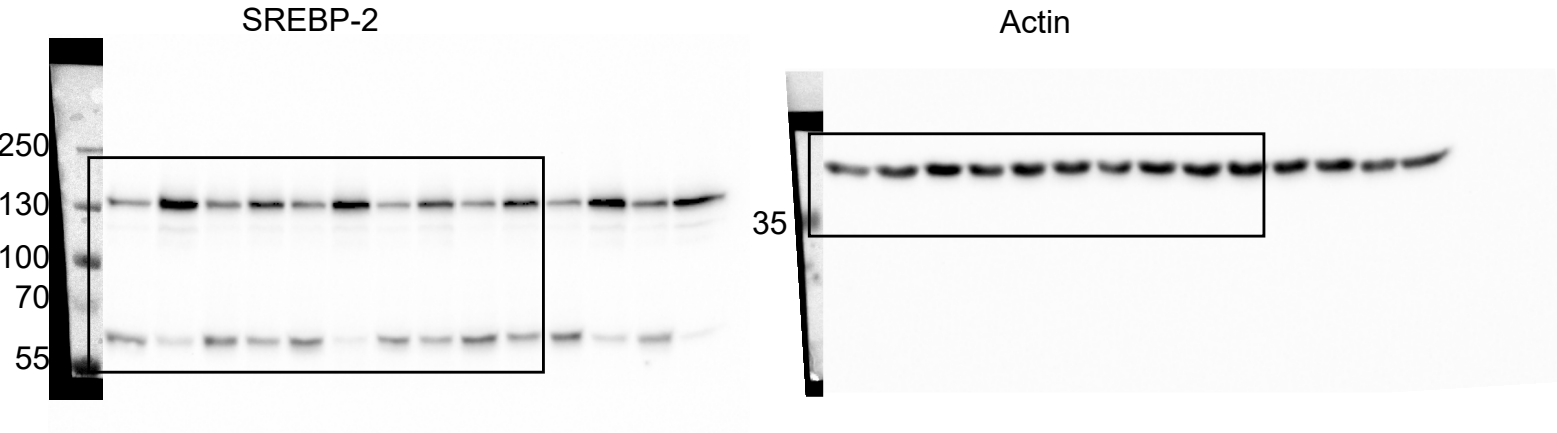

Supplement: Supplementary file 12 — Source Data for Figure 5 [file EMBJ-40-e106524-s008.pdf]

**Figure 6H**

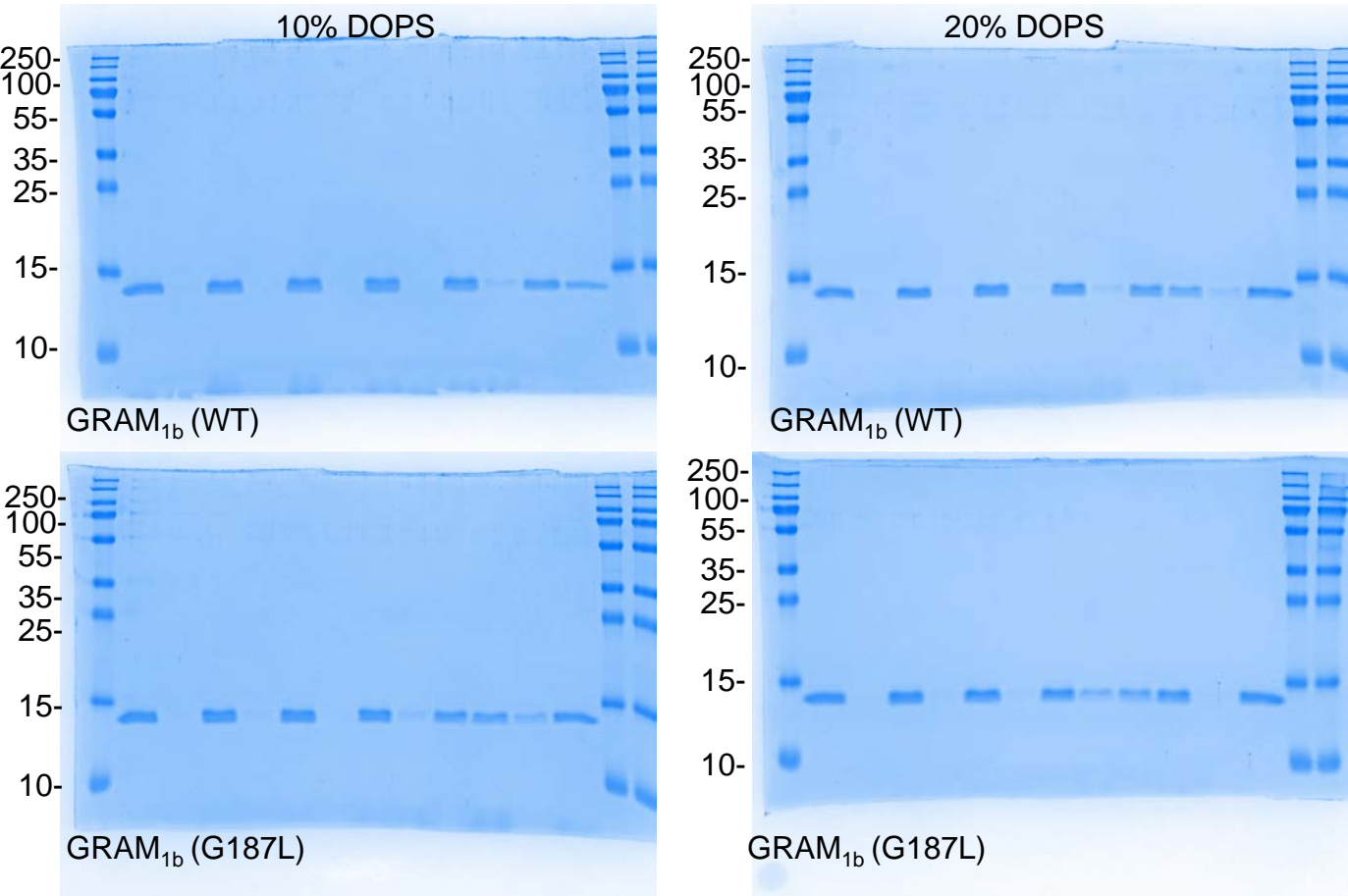

**Figure 6I**

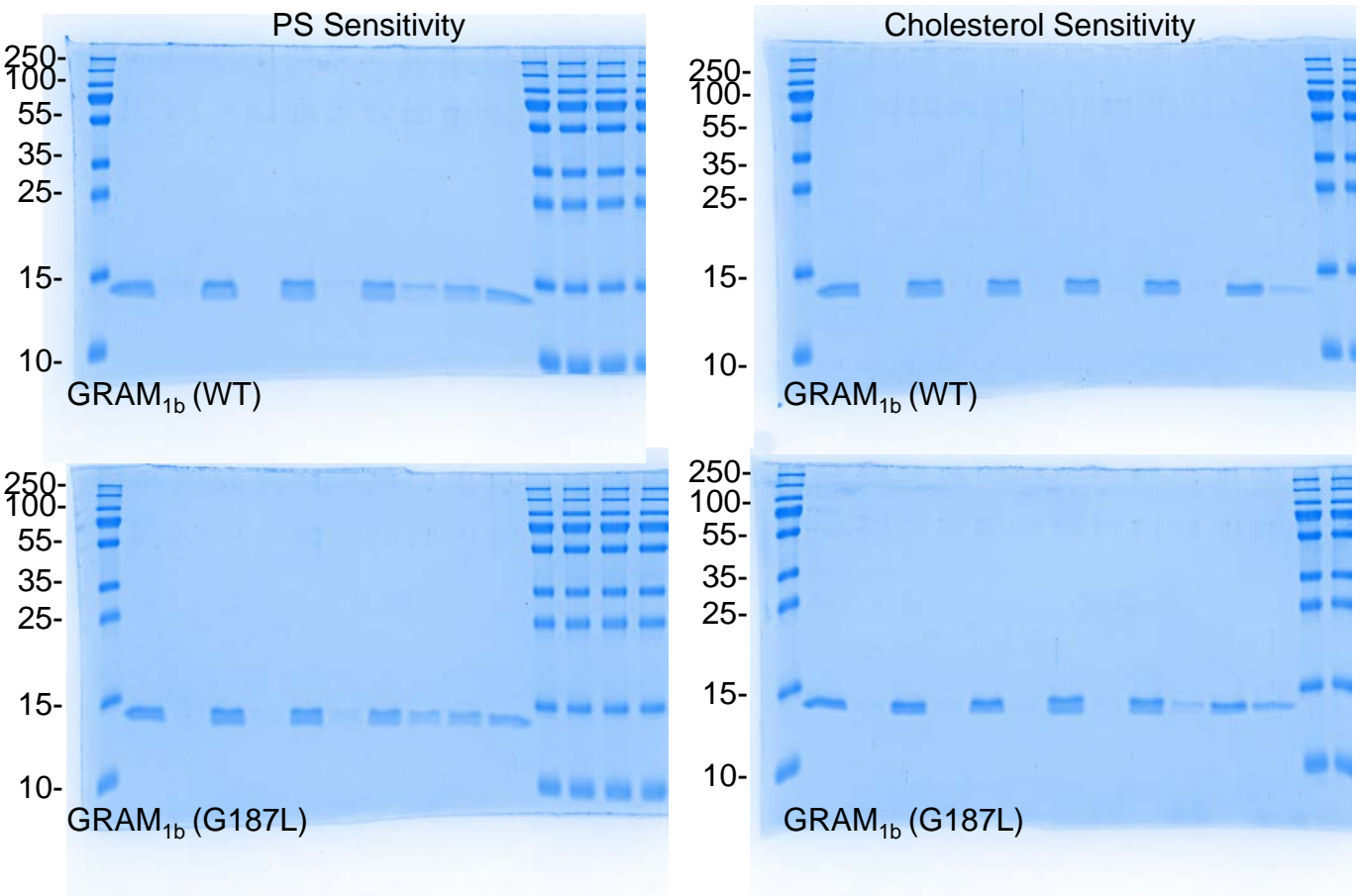

**Figure 6 Source Data**

Supplement: Supplementary file 13 — Source Data for Figure 6 [file EMBJ-40-e106524-s005.pdf]
