# Supplementary material for: Molecular basis of accessible plasma membrane cholesterol recognition by the GRAM domain of GRAMD1b
Source: EMBO J. 2021 Feb 19;40(6):e106524. doi: 10.15252/embj.2020106524 (PMC7957428; doi:10.15252/embj.2020106524)
Supplement: Supplementary file 10 — Source Data for Figure 2 [file EMBJ-40-e106524-s006.pdf]

Figure 2C

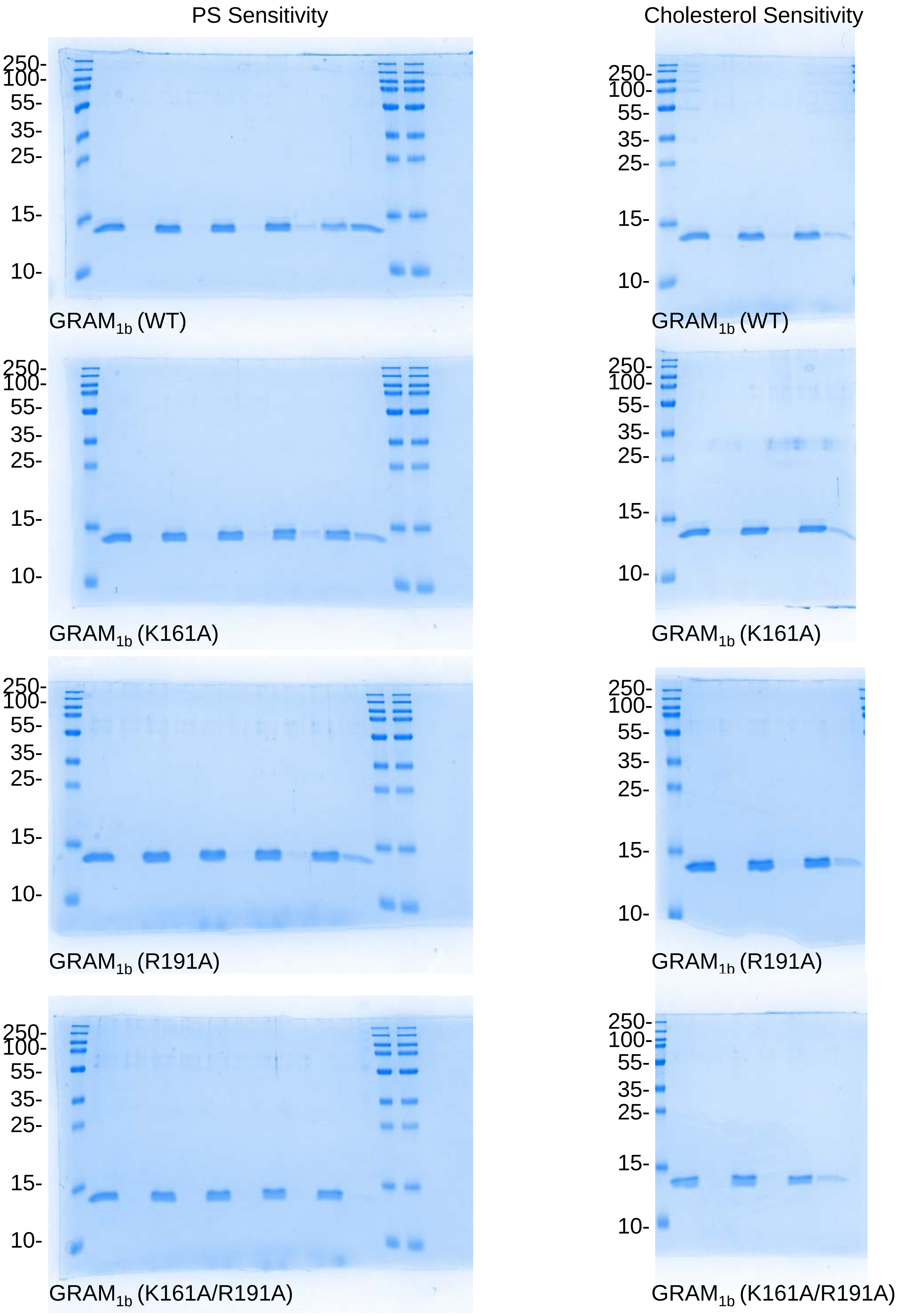

Figure 2 Source Data

Figure 2D

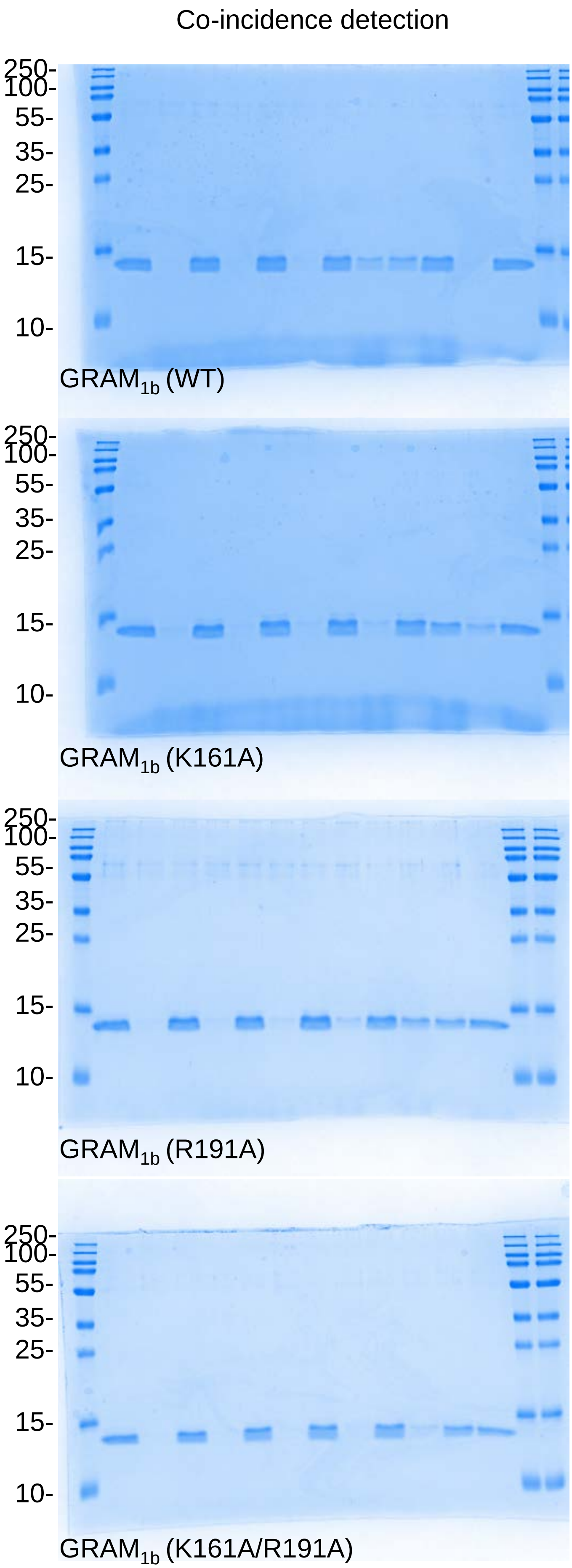

Figure 2E

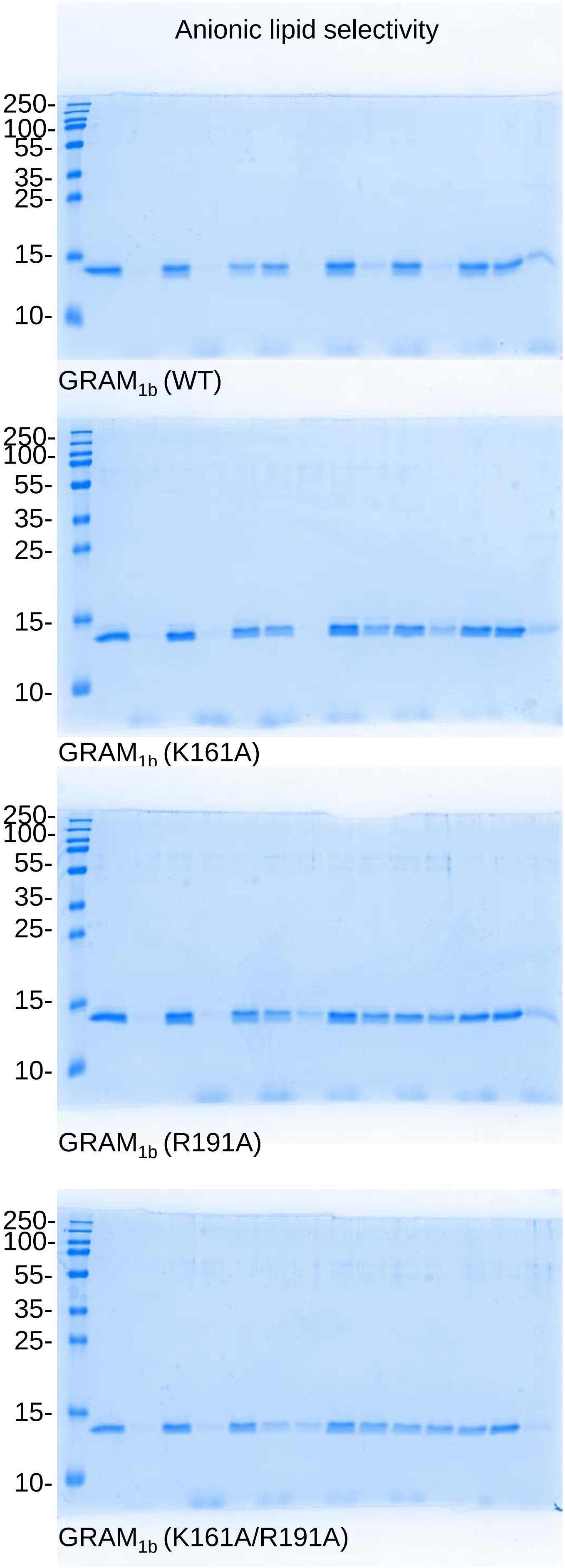

Figure 2 Source Data
